# Supplementary material for: A Comprehensive Review on Chemical Synthesis and Chemotherapeutic Potential of 3-Heteroaryl Fluoroquinolone Hybrids
Source: Antibiotics (Basel). 2023 Mar 21;12(3):625. doi: 10.3390/antibiotics12030625 (PMC10045242; doi:10.3390/antibiotics12030625)

Supplementary Materials

A Comprehensive Review on Chemical and Pharmacological Potential of 3-Heteroaryl Fluoroquinolone Hybrids

Halyna Hryhoriv [1], Sergiy M. Kovalenko [2,*], Marine Georgiyants [3], Lyudmila Sidorenko [1], Victoriya Georgiyants [1]

1 National University of Pharmacy; Pharmaceutical Chemistry Department
2 Karazin National University; Organic Chemistry Department
3 Kharkiv National Medical University; Department of Anesthesiology Intensive Therapy and Pediatrics Anesthesiology
* Correspondence: kovalenko.sergiy.m@gmail.com

Synthesis methods of 3-Heteroaryl Fluoroquinolone Hybrid Chemotypes

The chemotype is defined as 'a chemical structure motif or primary substructure that is common to a group of compounds' [Chao, Zhao, Dane, Huang, Ruyue, Li, Yida, Xu, Shimin, Su, Qiong, Gu, Jun, Xu. Identifying Novel Anti-Osteoporosis Leads with a Chemotype-Assembly Approach. *J. Med. Chem*. **2019**, 62, 12, 5885–5900 https://doi.org/10.1021/acs.jmedchem.9b00517]

[25] Lihumis, H. S., Al Talebi, Z. A., Khaleel, A. K. Synthesis and Identification of Some New Heterocyclic Compounds for Levofloxacin Drug Derivatives with Evaluating of Their Biological Efficiency and Antioxidant Activity. *Journal of Medicinal and Chemical Sciences*, **2022**; 5(4): 596-606. doi: 10.26655/JMCHEMSCI.2022.4.15 1_84 2022:956046

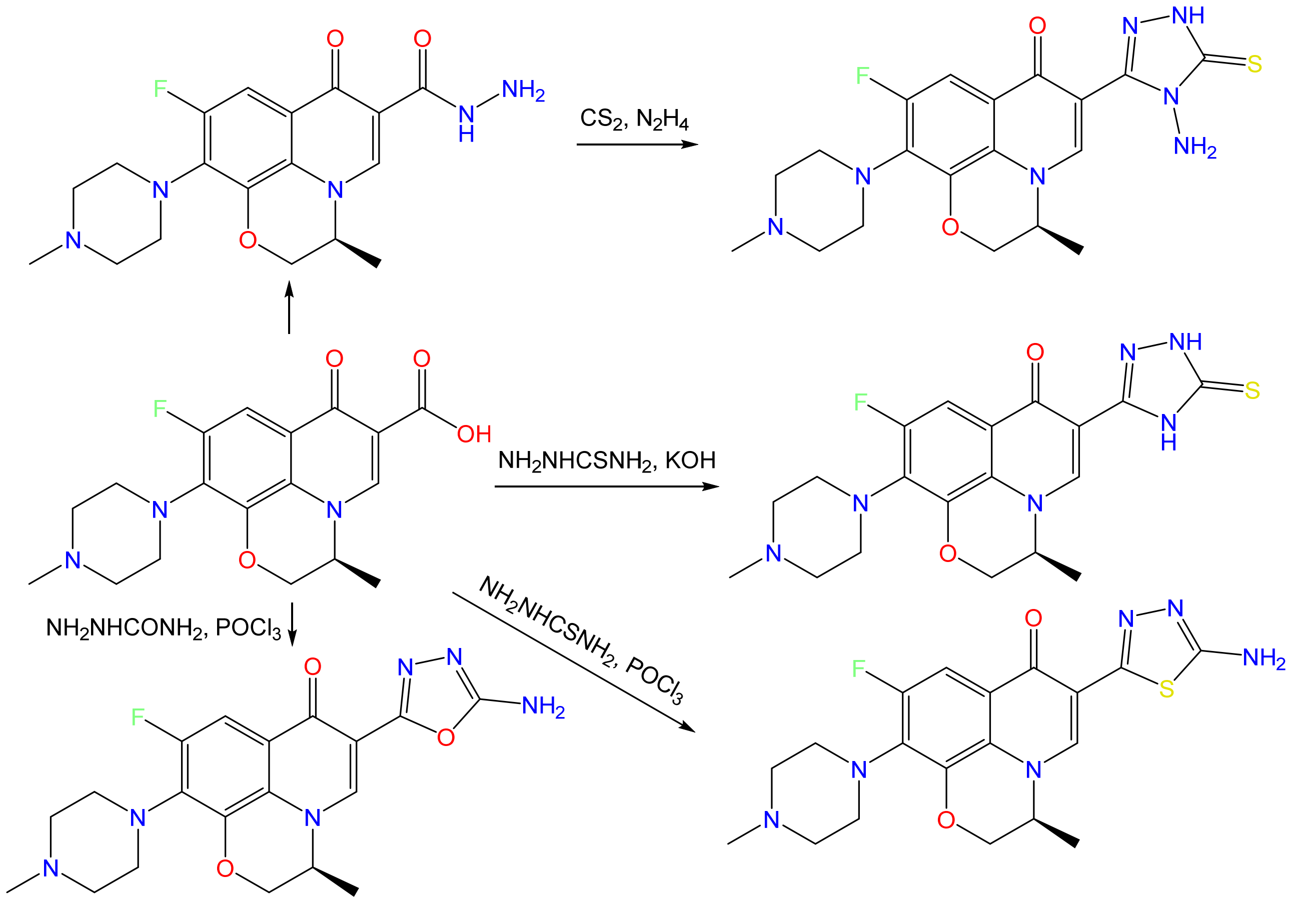

[26] Arshad, M., Khan, M.S., Nami, S.A. Norfloxacin Analogues: Drug Likeness, Synthesis, Biological, and Molecular Docking Assessment. *Russ J Bioorg Chem,* **2021,** 47, 483–495. https://doi.org/10.1134/S1068162021020047

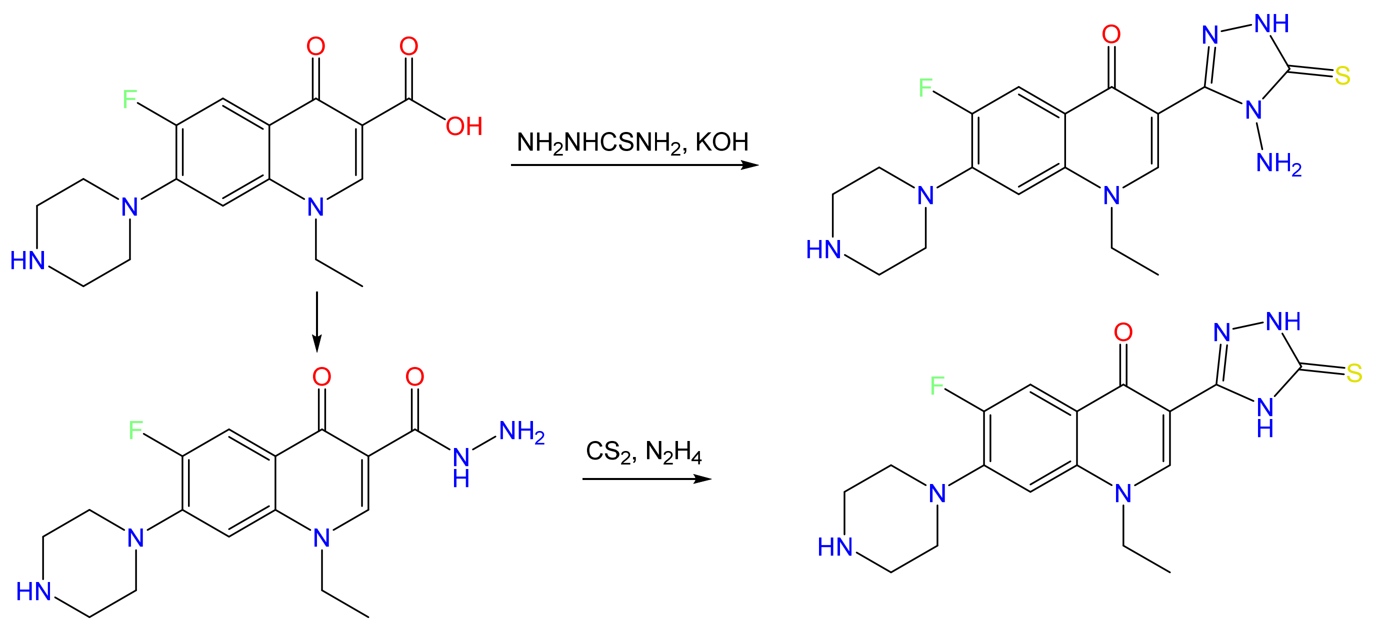

[27] Li, Tao; Gao, Liu-zhou; Xie, Yu-suo; Hu, Guo-qiang; Huang, Wen-long Synthesis and antitumor activity fluoroquinolon-3-yloxadiazole sulfanylacetylhydrazone derivatives. *Zhongguo Yaoxue Zazhi (Beijing, China*), **2014**, 49(24), 2206-2209

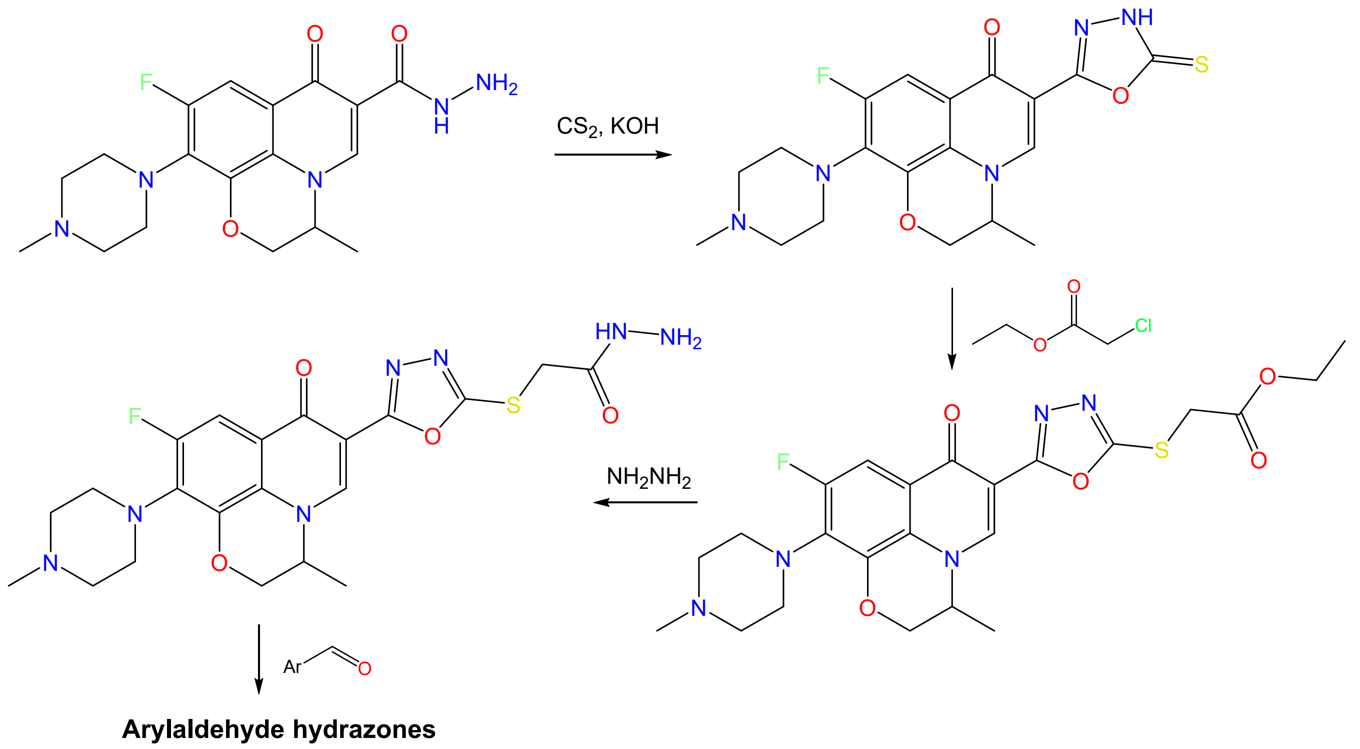

[28] Chen Yinsheng, Wang Guoqiang, Duan Nannan, Cao Tieyao, Wen Xiaoyi, Yin Jun, Wang Wei, Xie Songqiang, Huang Wenlong, Hu Guoqiang. Synthesis and Antitumor Activity of Fluoroquinolone C3-Isostere Derivatives: Oxadiazole Mannich Base Derivatives. *Chinese Journal of Applied Chemistry*, **2012,** 29(11), p. 1246-1250. doi: 10.3724/SP.J.1095.2012.00537.

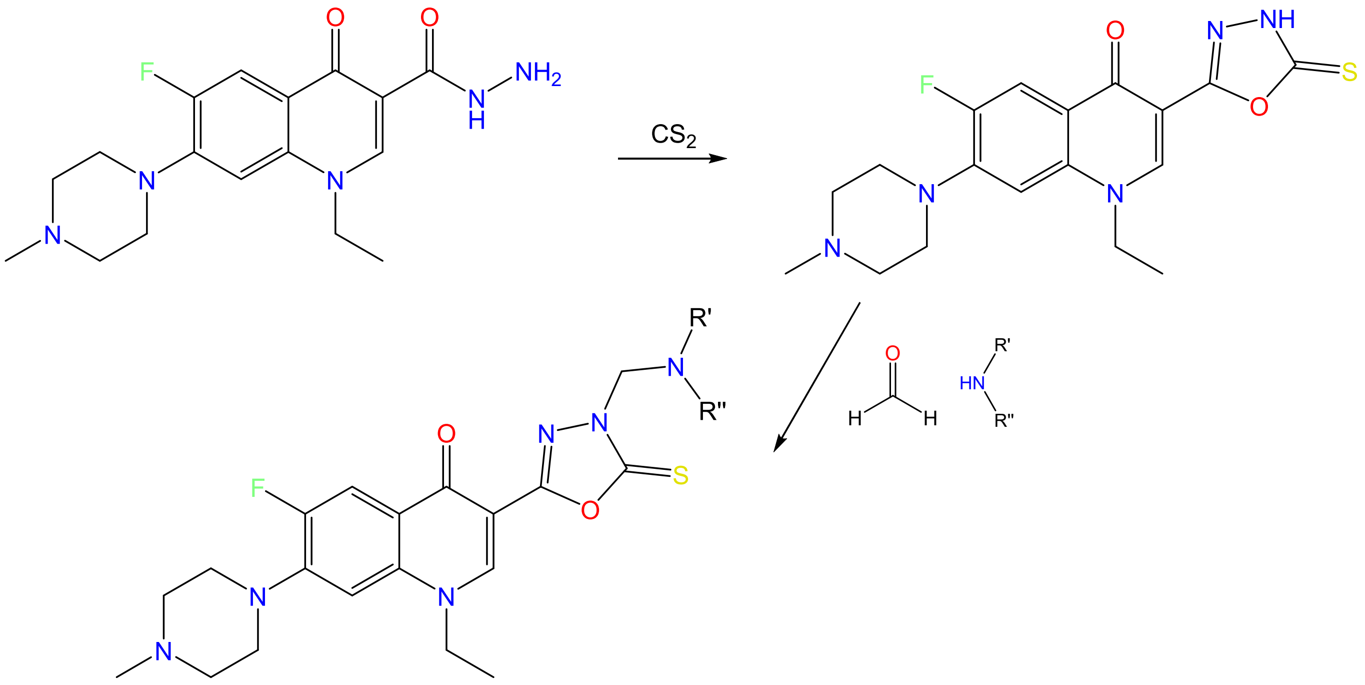

[29] Khan, Nawaz Mohammed; Kumar, Pawan; Hemanth, Sudheer Kumar K.; Bharath, Rathna Kumar P. Synthesis, molecular bioinformatics modelling, and antimicrobial evaluation of some novel oxadiazole fluoroquinolone derivatives. *Asian Journal of Pharmaceutical and Clinical Research*, **2022**, 15(1), 40-46

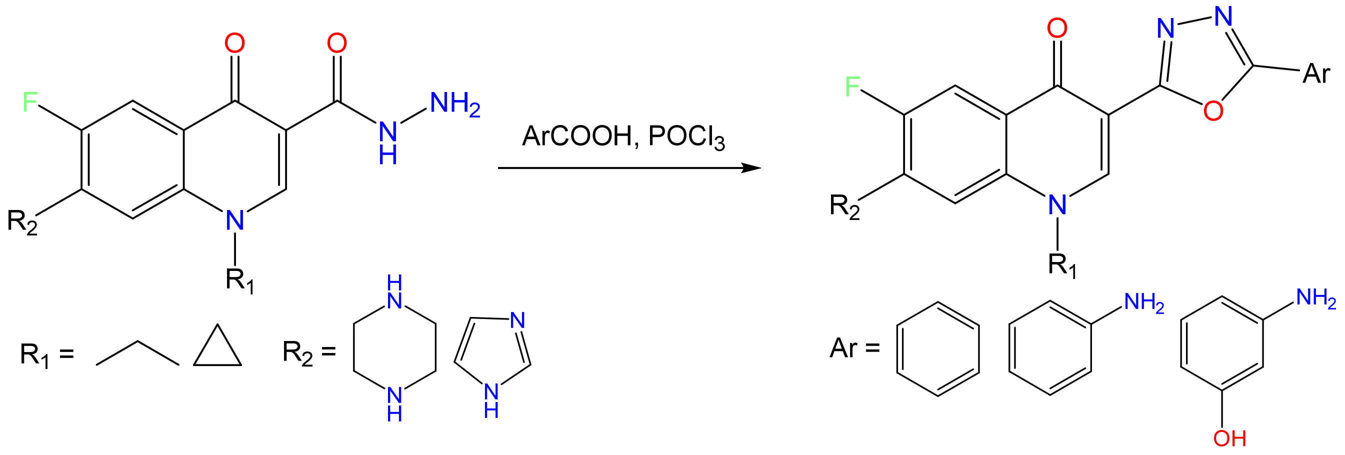

[30] Allaka, Tejeswara Rao; Katari, Naresh Kumar; Jonnalagadda, Sreekantha B.; Malkhed, Vasavi; Anireddy, Jaya Shree Design, Synthesis and Biological Evaluation of Novel Heterocyclic Fluoroquinolone Citrate Conjugates as Potential Inhibitors of Topoisomerase IV: A Computational Molecular Modeling Study. *Current Drug Discovery Technologies*, **2020**, 17(6)

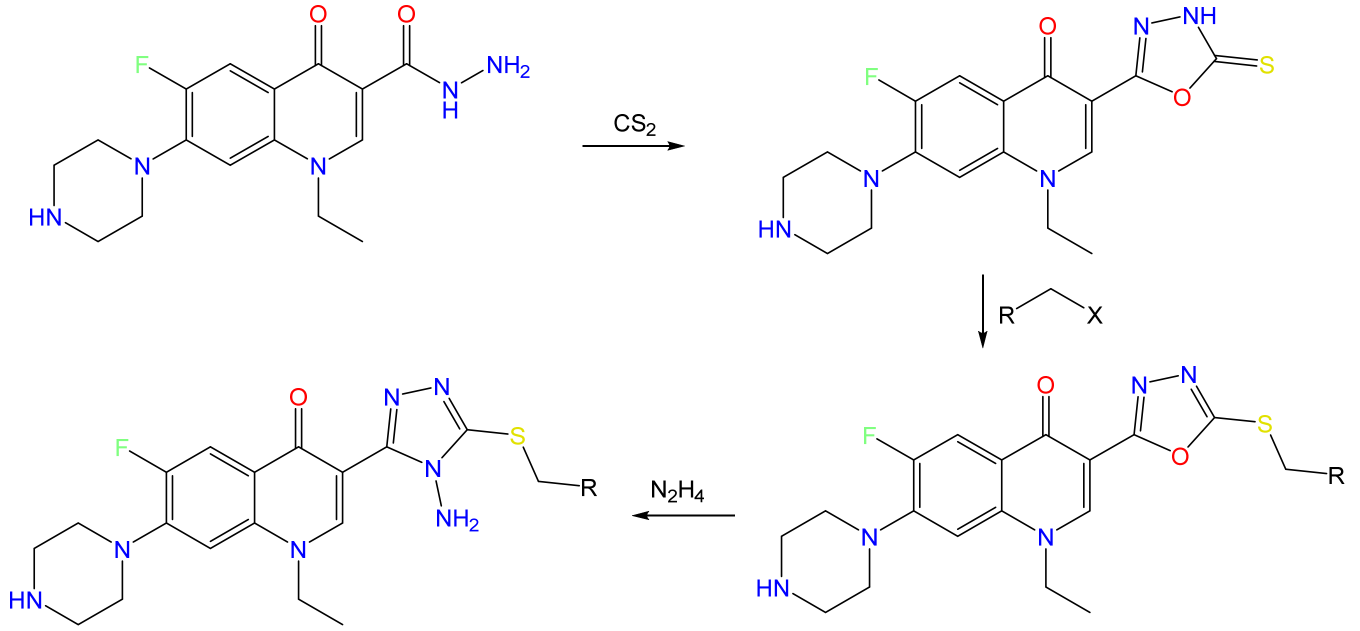

[31] Allaka, Tejeswara Rao, Kummari, Bhaskar; Polkam, Naveen; Kuntala, Naveen; Chepuri, Kalyani; Anireddy, Jaya Shree Novel heterocyclic 1,3,4-oxadiazole derivatives of fluoroquinolones as a potent antibacterial agent: Synthesis and computational molecular modeling. *Molecular Diversity*, **2022**, 26(3), 1581-1596

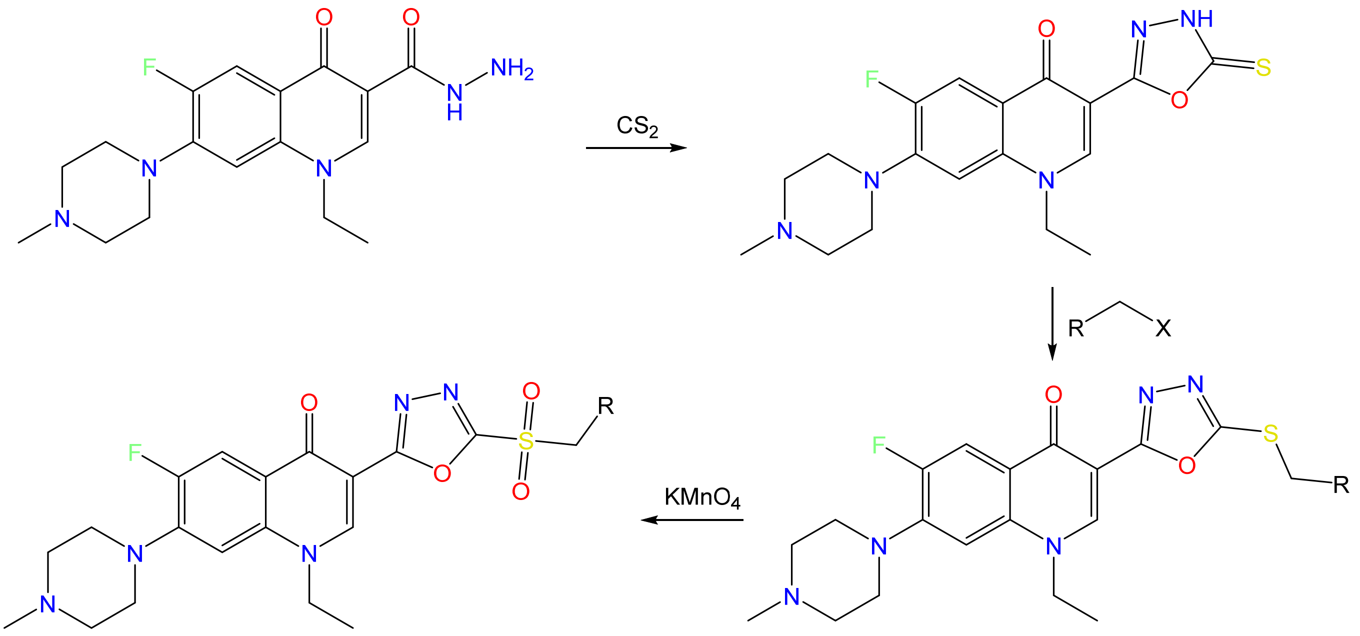

[32] Niveditha, Nakka; Begum, Munnisa; Prathibha, Duvvala; Sirisha, Kalam; Mahender, Porika; Chitra, Chandrashekar; Rao, Vedula Rajeswar; Reddy, Vanga Malla; Achaiah, Garlapati. Design, synthesis and pharmacological evaluation of some C3 heterocyclic-substituted ciprofloxacin derivatives as chimeric antitubercular agents. *Chemical & Pharmaceutical Bulletin*, **2020**, 68(12), 1170-1177

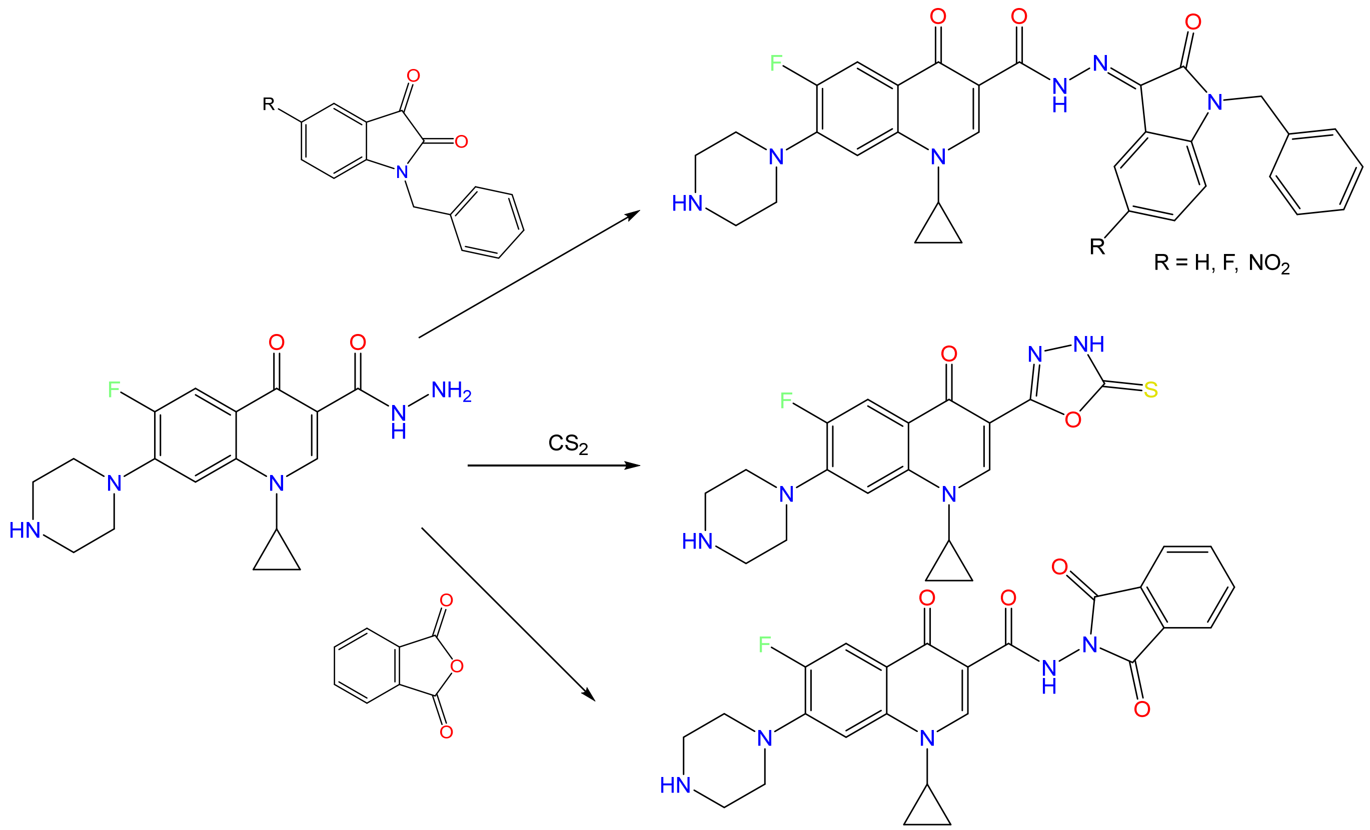

[33] Singhai, Anuj; Gupta, M. K. Synthesis, characterization and biological evaluation of substituted 1,3,4-oxadiazole derivative: derived from ciprofloxacin. *Asian Journal of Pharmaceutical and Clinical Research*, **2019**, 12(9), 205-209

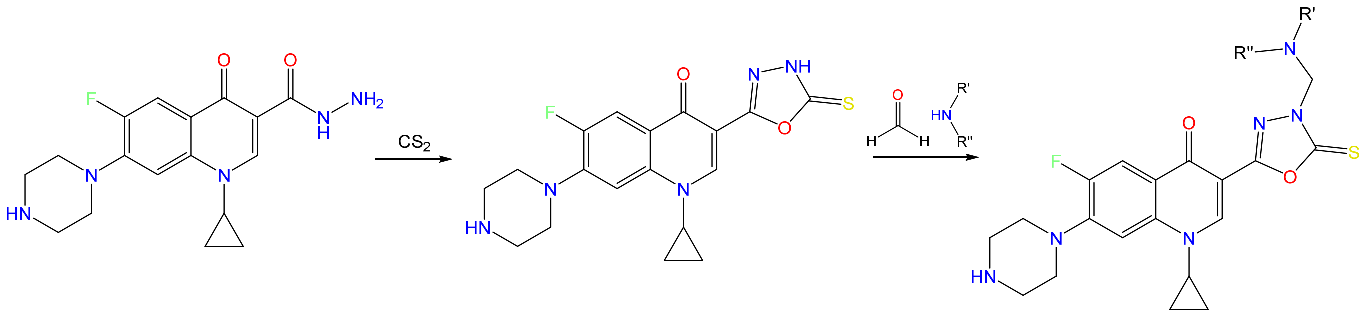

[34] Wu, Shu-min; Hou, Ping-ping; Wen, Jing; Hu, Guo-qiang Synthesis and antitumor activity of oxadiazolone thione Mannich-base derivatives of enrofloxacin. *Shenyang Yaoke Daxue Xuebao*, **2015**, 32(11), 840-843, 858

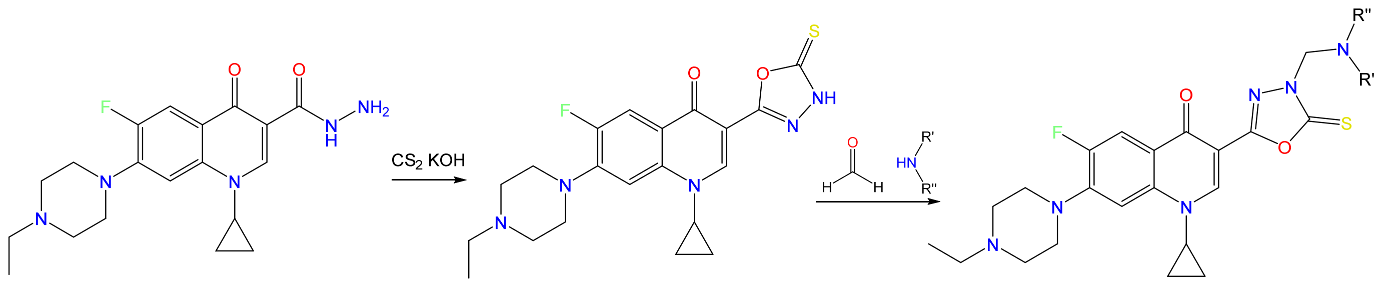

[35] Shang, Hui-jie; Yan, Qiang; Wu, Shu-min; Ni, Li-li; Huang, Wen-long; Hu, Guo-qiang Synthesis and antitumor activity of novel oxadiazole derivatives of enrofloxacin. *Shenyang Yaoke Daxue Xuebao,* **2015**, 32(8), 604-608

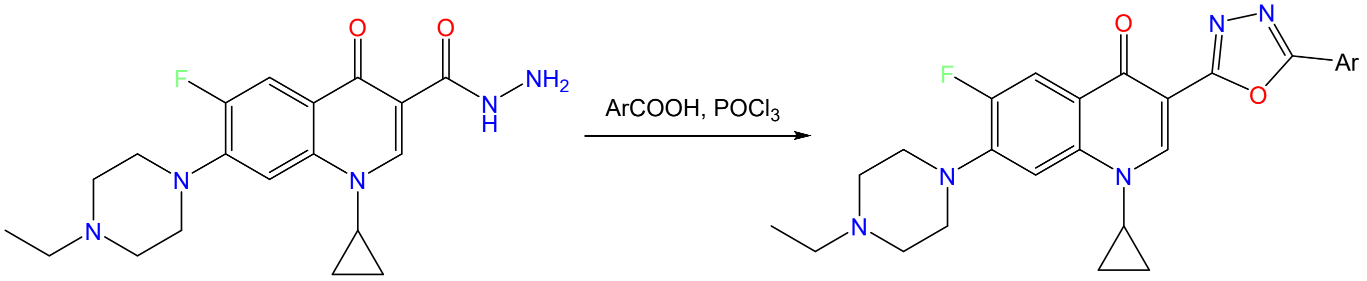

[36] Gao, Liu-zhou; Xie, Yu-suo; Li, Tao; Huang, Wen-long; Hu, Guo-qiang Synthesis, antitumor activity and SAR of C-3 oxadiazole sulfanylacetylhydrazone-substituted fluoroquinolone analogues. *Yaoxue Xuebao*, **2014**, 49(12), 1694-1698

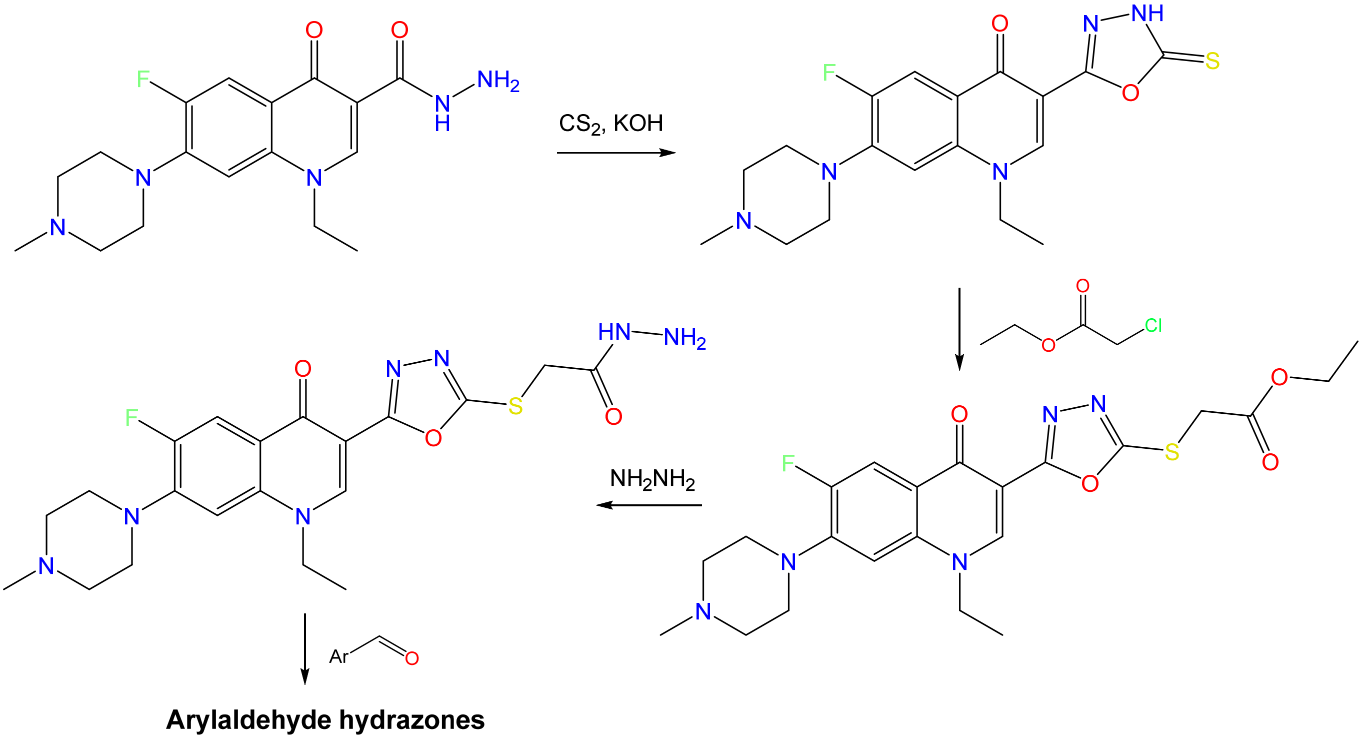

[37] Hou, Li-li; Yin, Jun; Wang, Wei; Xie, Song-qiang; Huang, Wen-long; Hu, Guo-qiang Synthesis and antitumor activity of fluoroquinolone C-3 heterocycles-oxadiazole derivatives (II). *Zhongguo Yaoxue Zazhi (Beijing, China),* **2013**, 48(14), 1194-1196

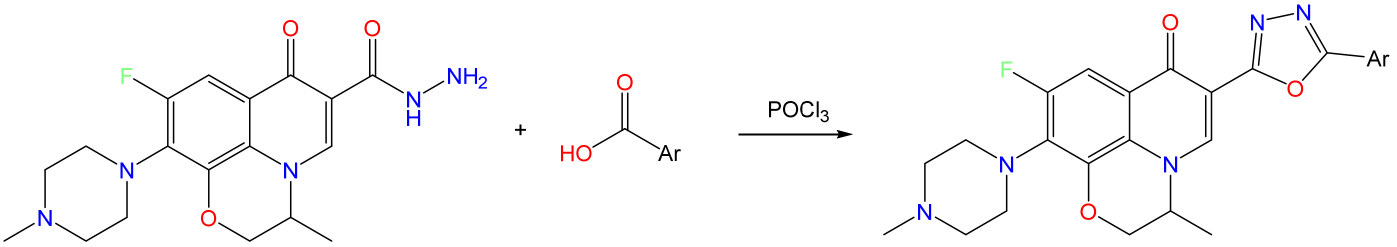

[38] Hu, Guo-qiang; Wang, Guo-qiang; Duan, Nan-nan; Wen, Xiao-yi; Cao, Tie-yao; Xie, Song-qiang; Huang, Wen-long Design, Synthesis and Antitumor Activity of Fluoroquinolone C3 Heterocyclic Bis-oxadiazole Methylsulfide Derivatives Derived from Levofloxacin. *Chemical Research in Chinese Universities*, **2012**, 28(6), 980-984

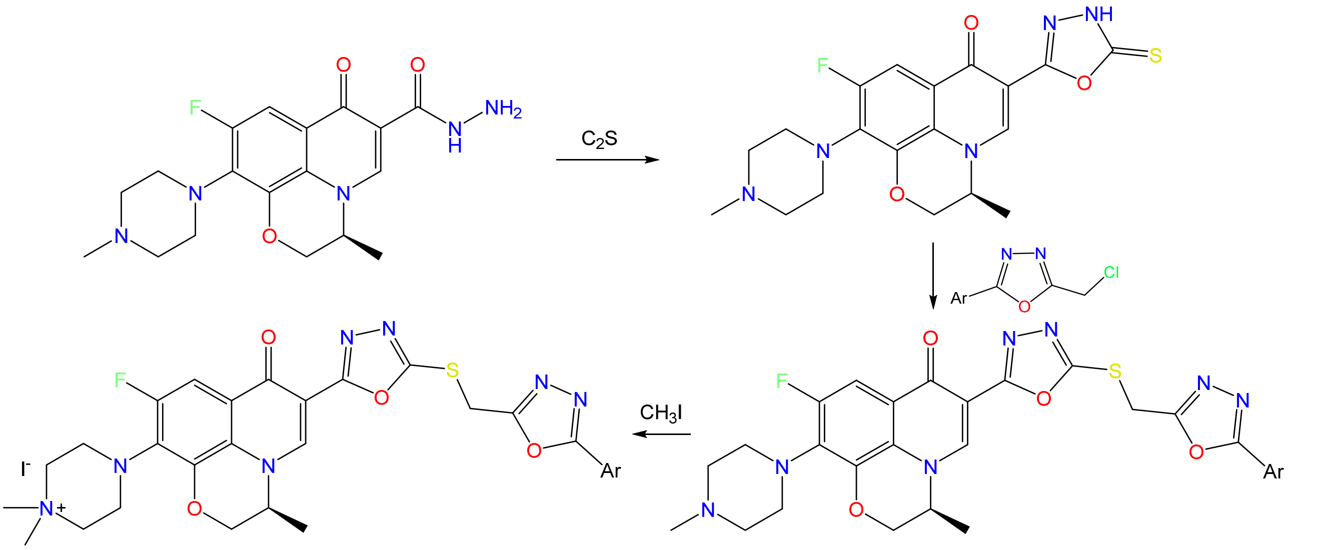

[39] Wang, Guoqiang; Duan, Nannan; Cao, Tieyao; Wen, Xiaoyi; Yin, Jun; Wang, Wei; Xie, Songqiang; Huang, Wenlong; Hu, Guoqiang Antitumor fluoroquinolone C3-isostere derivatives(I) - synthesis and activity of bis-oxadiazole methyl-sulfide derivatives. *Yingyong Huaxue*, **2012**, 29(7), 769-774

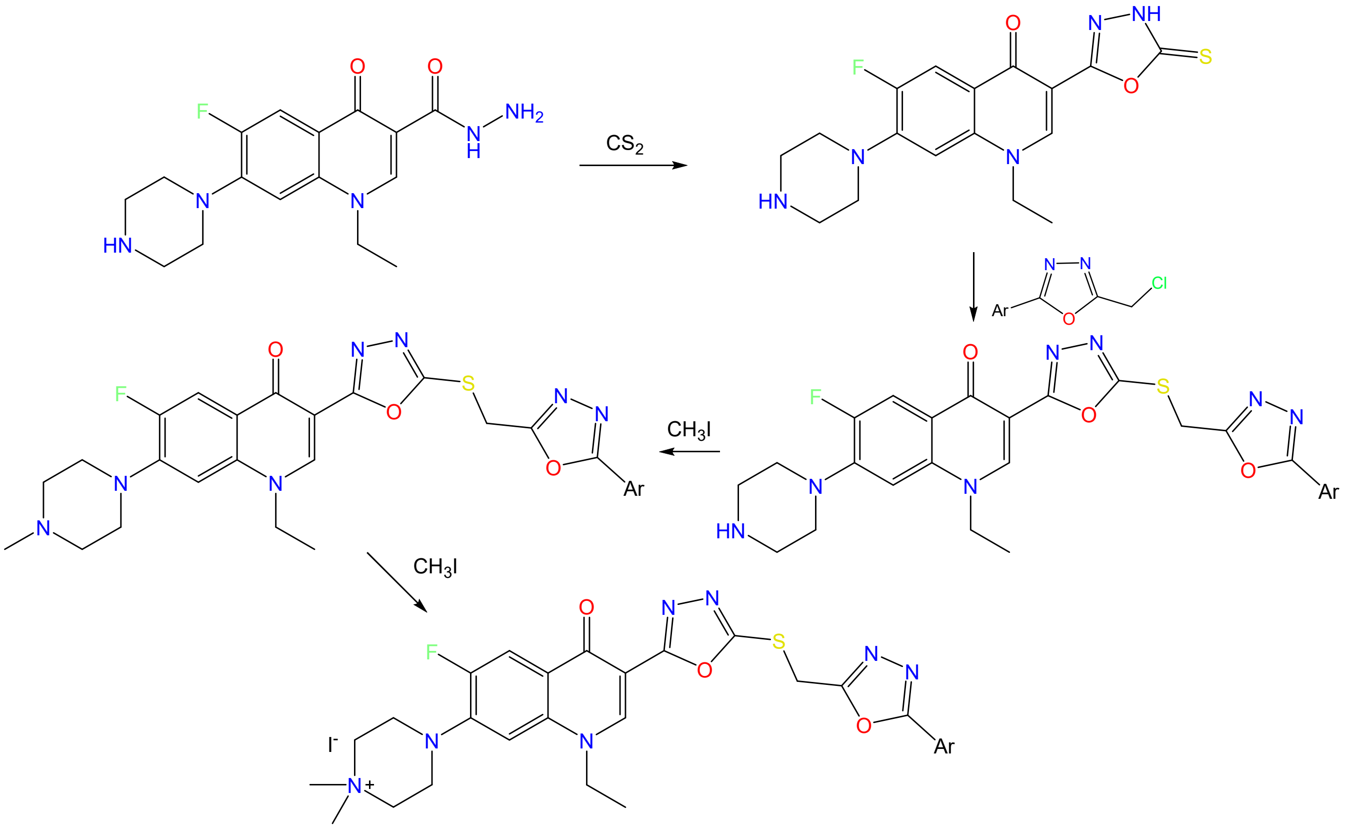

[41] Xie, Yusuo; Gao, Liuzhou; Yan, Qiang; Wu, Shumin; Ni, Lili; Liu, Yingjie; Huang, Wenlong; Hu, Guoqiang Synthesis and antitumor activity of fluoroquinolon-3-yl s-triazole sulfide ketone thiosemicarbazone derivatives of ofloxacin. *Yingyong Huaxue*, **2016**, 33(1), 25-31

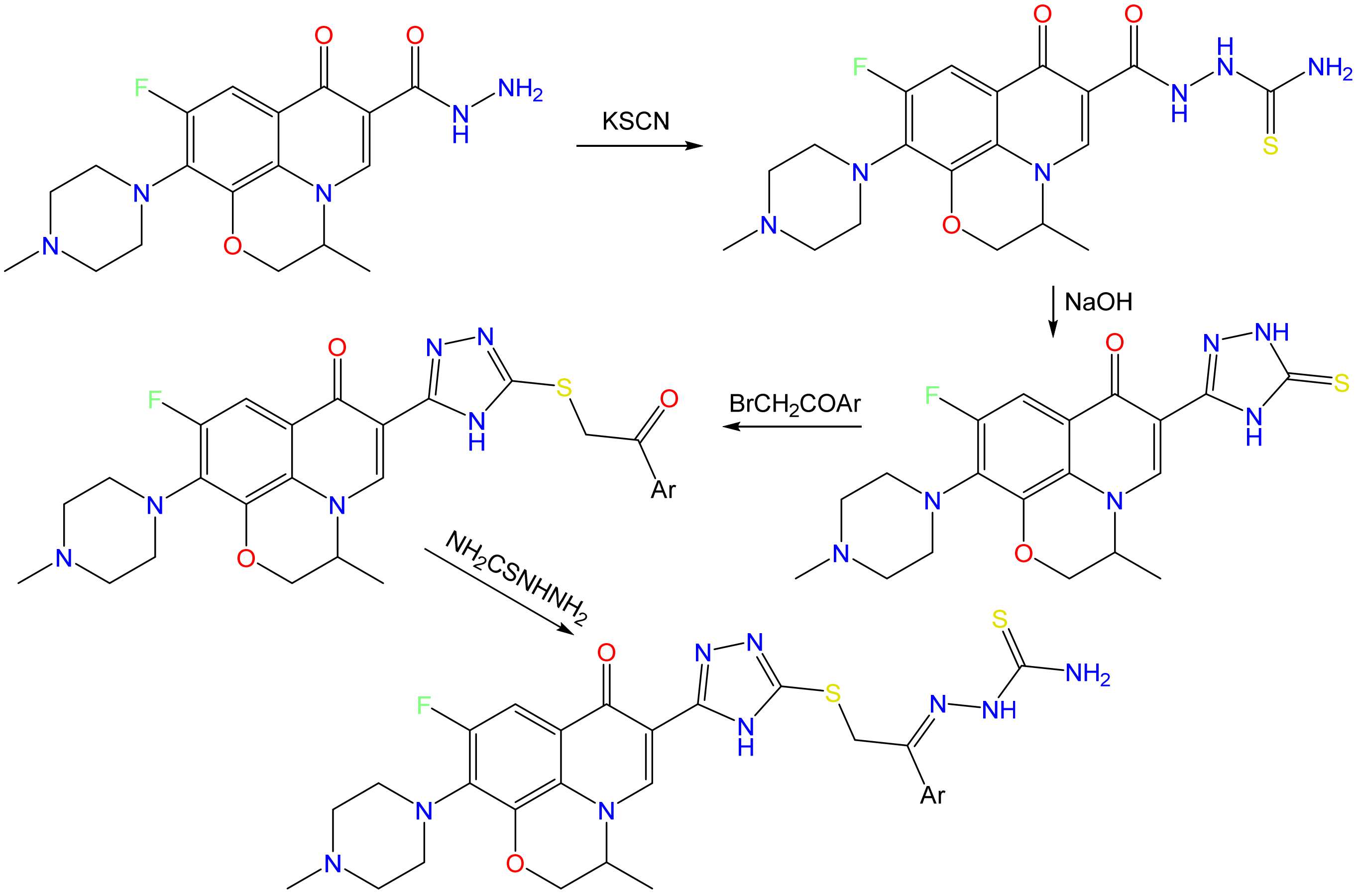

[42] Sun, Yusheng; Xu, Qiuju; Hou, Lili; Yue, Xibo; Wu, Zhaofeng; Huang, Wenlong; Xie, Songqiang; Hu, Guoqiang Synthesis and antitumor activities of fluoroquinolone C-3 isosteres(IV):s-triazole-oxadiazole methylsulfide Mannich-base derivatives. *Zhongguo Yaoke Daxue Xuebao*, **2014,** 45(1), 39-42

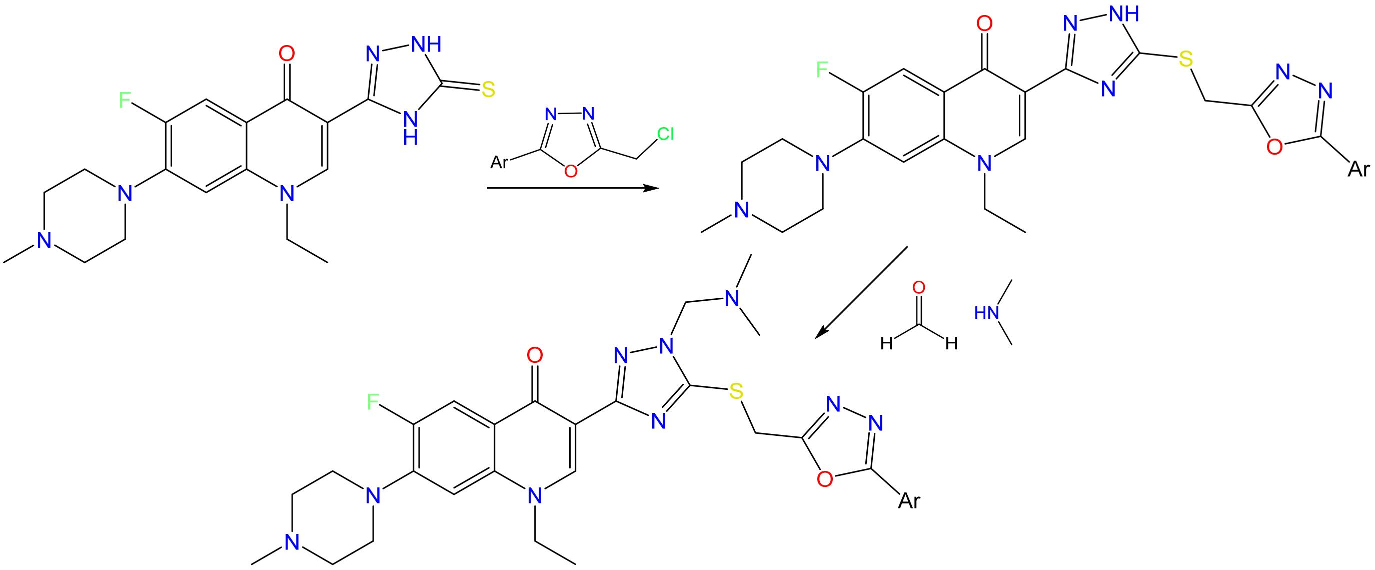

[43] Xu, Qiuju; Hou, Lili; Wu, Zhaofeng; Yue, Xibo; Hu, Guoqiang; Huang, Wenlong Synthesis and antitumor activity of fluoroquinolone C-3 isostere III: s-triazole oxadiazole methylsulfide derivatives from pefloxacin. *Zhongguo Yaoke Daxue Xuebao*, **2013**, 44(6), 511-514

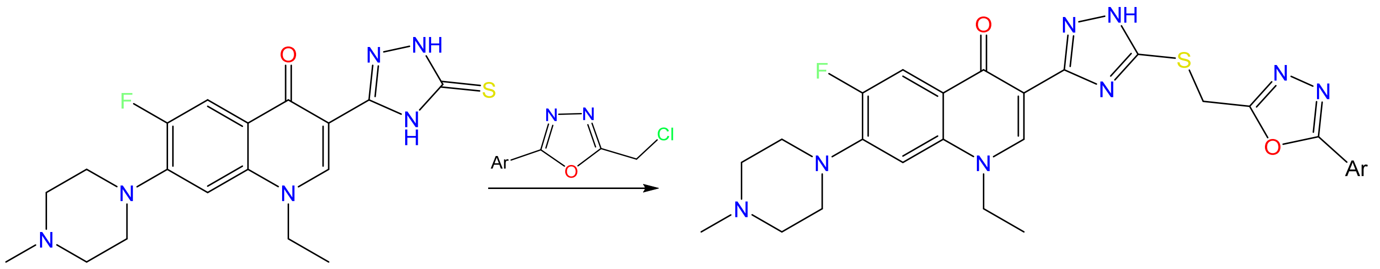

[44] Xu, Qiu-ju; Hou, Li-li; Wu, Zhao-feng; Yue, Xi-bo; Xie, Song-qiang; Huang, Wen-long; Hu, Guo-qiang Synthesis and antitumor evaluation of fluoroquinolone C3 s-triazoie oxadiazole methylsulfide derivatives of ofloxacin. *Zhongguo Yaoxue Zazhi (Beijing, China)*, **2014**, 49(7), 609-612

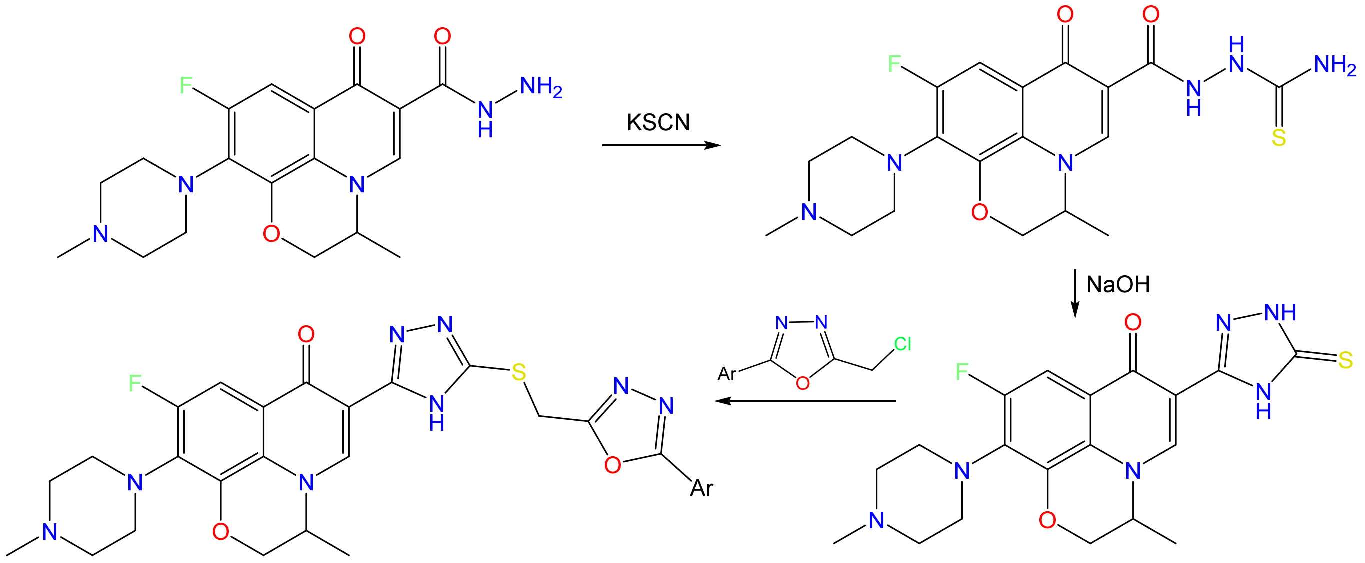

[45] Hu, Guoqiang; Wang, Guoqiang; Duan, Nannan; Wen, Xiaoyi; Cao, Tieyao; Xie, Songqiang; Huang, Wenlong Design, synthesis and antitumor activities of fluoroquinolone C-3 heterocycles (IV): s-triazole Schiff-Mannich bases derived from ofloxacin. *Acta Pharmaceutica Sinica B*, **2012**, 2(3), 312-317

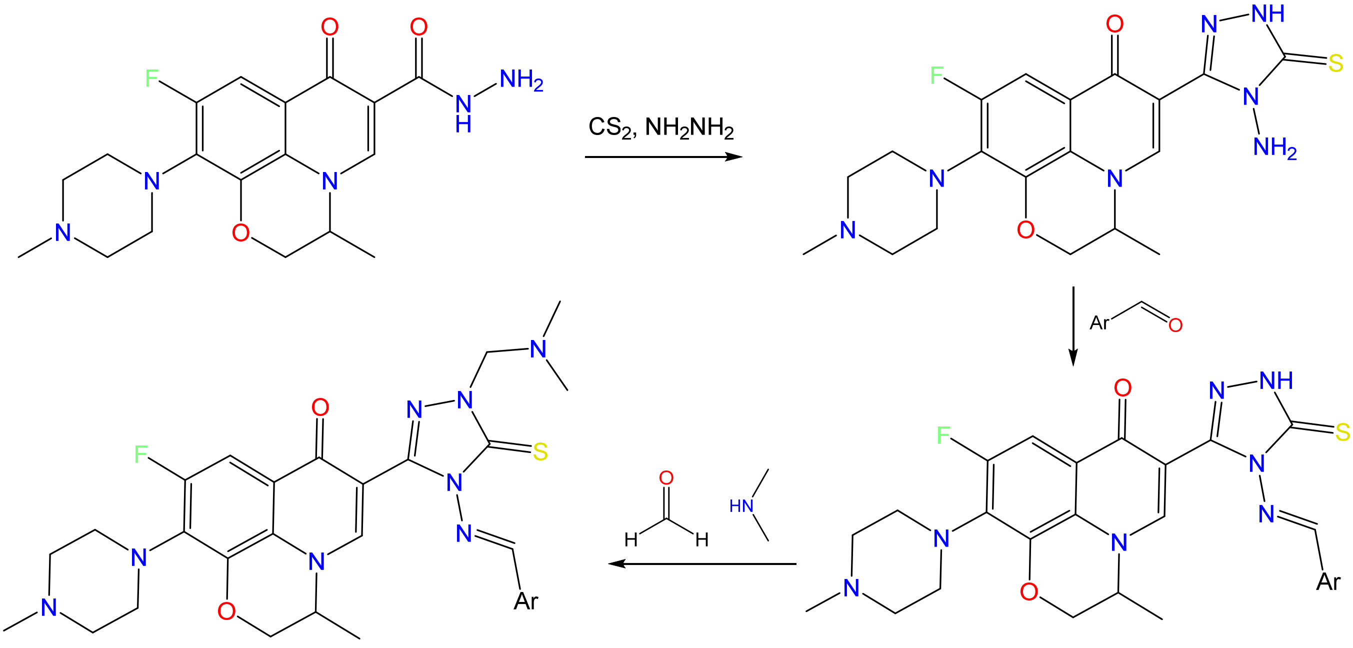

[46] Jubie, S.; Prabitha, P.; Rajesh Kumar, R.; Kalirajan, R.; Gayathri, R.; Sankar, S.; Elango, K. Design, synthesis, and docking studies of novel ofloxacin analogues as antimicrobial agents. *Medicinal Chemistry Research*, **2012**, 21(7), 1403-1410

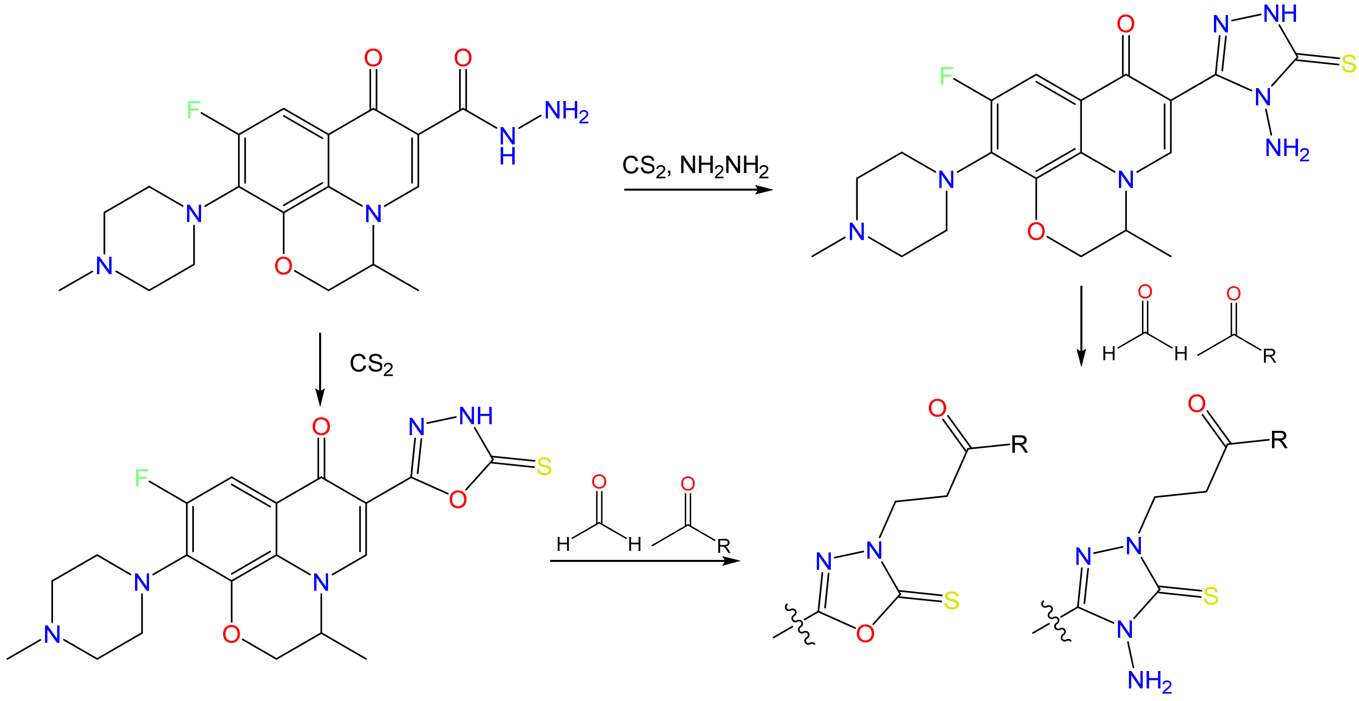

[47] Hu, Guo-Qiang; Wu, Xiao-Kui; Xie, Song-Qiang; Du, Gang-Jun; Huang, Wen-Long; Zhang, Hui-Bin Synthesis and bioactivity of water-soluble fused s-triazolothiadiazole systems (II): fluoroquinolone piperazine derivatives. *Huaxue Xuebao,* **2008**, 66(19), 2157-2162

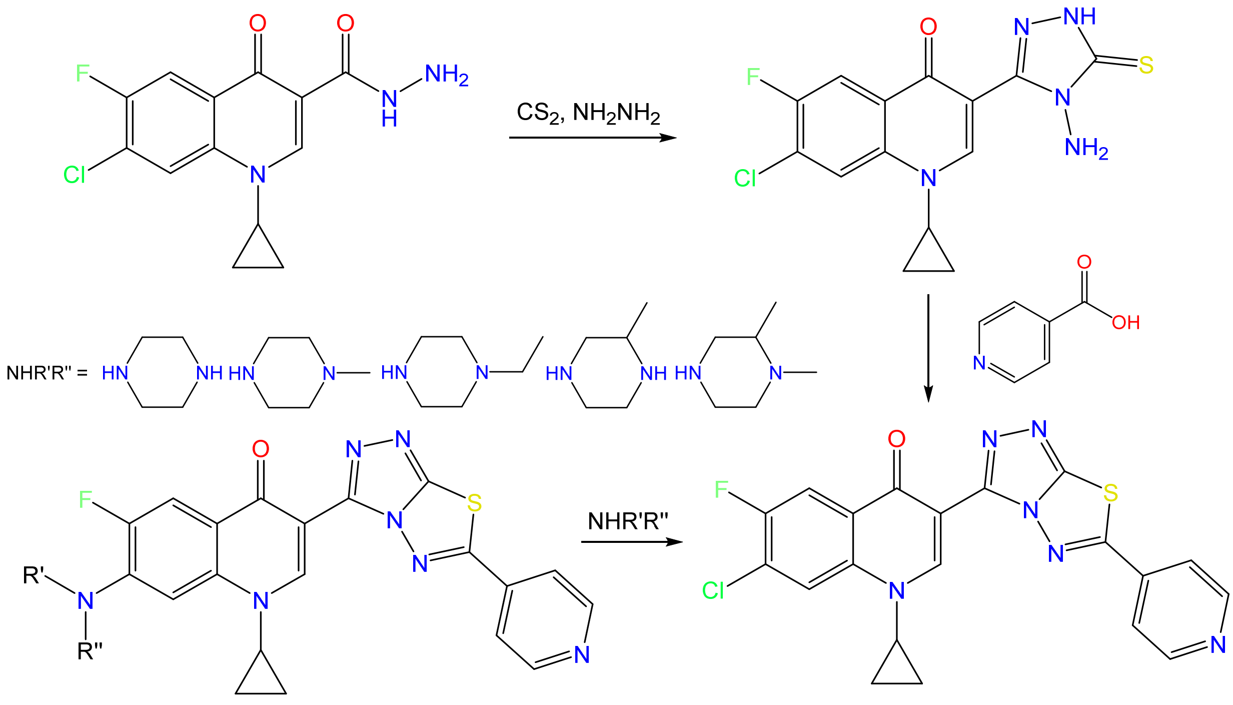

[48] Li, Yuanyuan, Zhang, Chengxia, Huang, Wenlong, Chen, Chaoran, Hu, Guoqiang. Synthesis and Antitumor Activities of C-3 Thiazolotriazole Unsaturated Ketone Derivatives of Levofloxacoin. *Chinese Journal of Applied Chemistry,* **2019**, 36(6): 671-676.

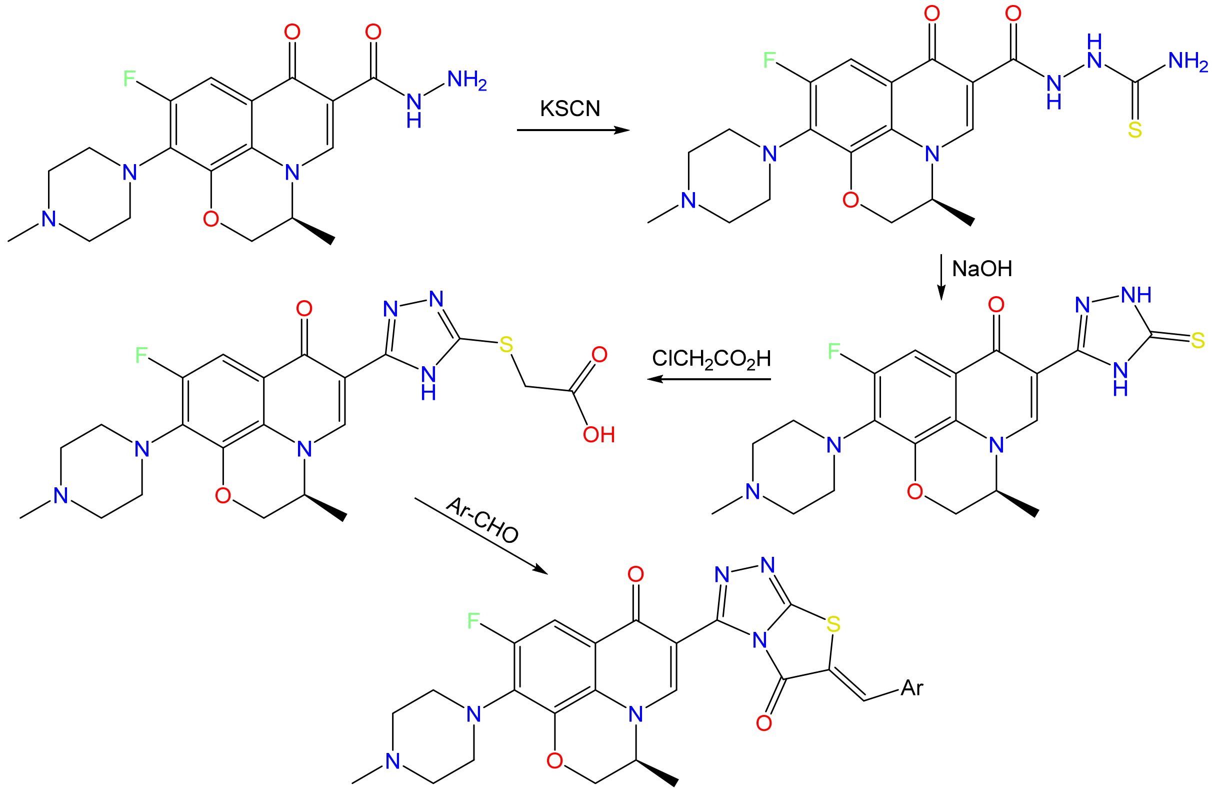

[49] Xie, Songqiang; Chen, Yinsheng; Wang, Guoqiang; Duan, Nannan; Wen, Xiaoyi; Cao, Tieyao; Yin, Jun; Wang, Wei; Hu, Guoqiang; Huang, Wenlong Synthesis and antitumor evaluation of s-triazolothiadiazines and pyrazolo s-triazoles derived from ciprofloxacin. *Yaoxue Xuebao*, **2012**, 47(1), 66-71

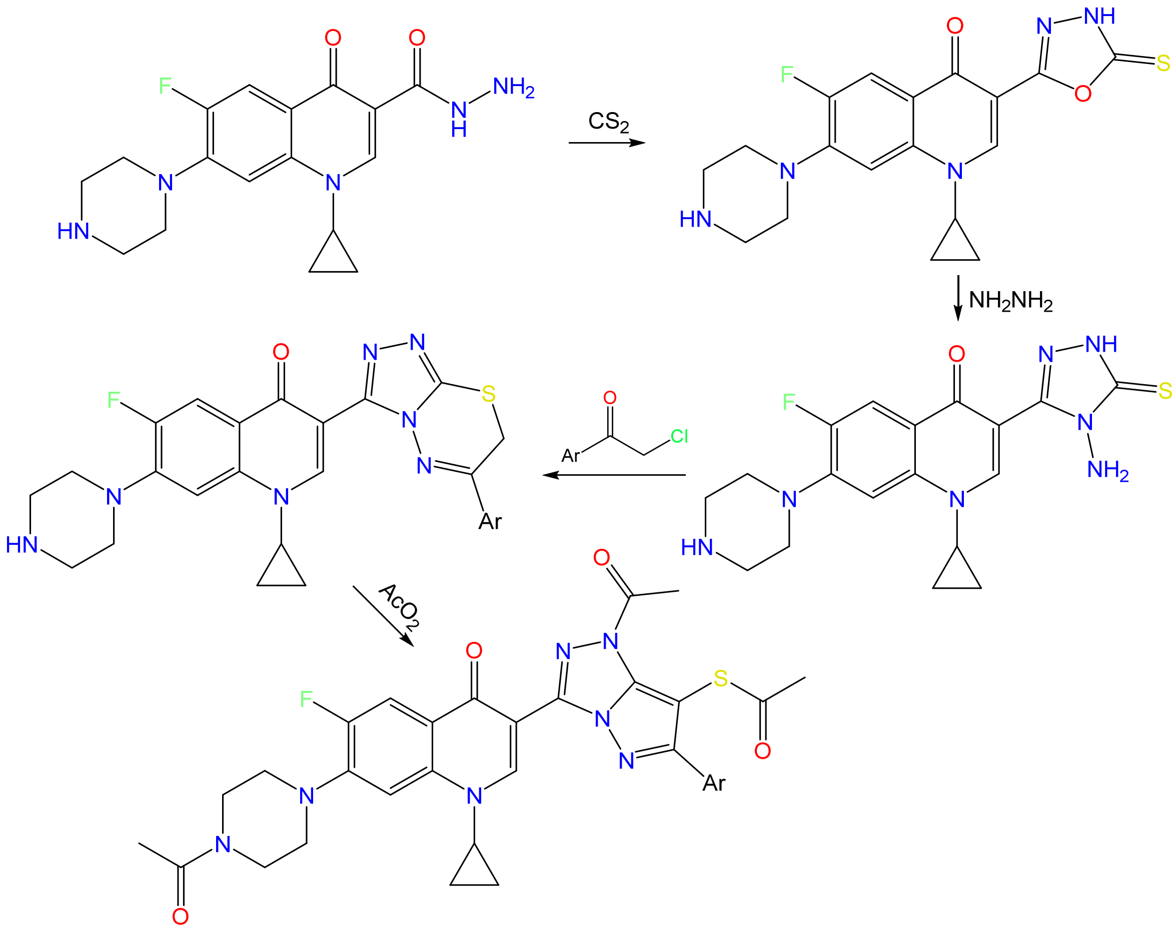

[50] Hu, Guoqiang; Zhang, Zhongquan; Wang, Haiyan; Wu, Xiaokui; Wang, Xin; Du, Gangjun; Xie, Songqiang; Huang, Wenlong; Zhang, Huibin Synthesis and antitumor activity of fluoroquinolon-3-yl fused heterocyclic systems (III): s-triazolothiadiazinone derivatives derived enrofloxacin. *Huaxue Xuebao*, **2009,** 67(22), 2592-2596

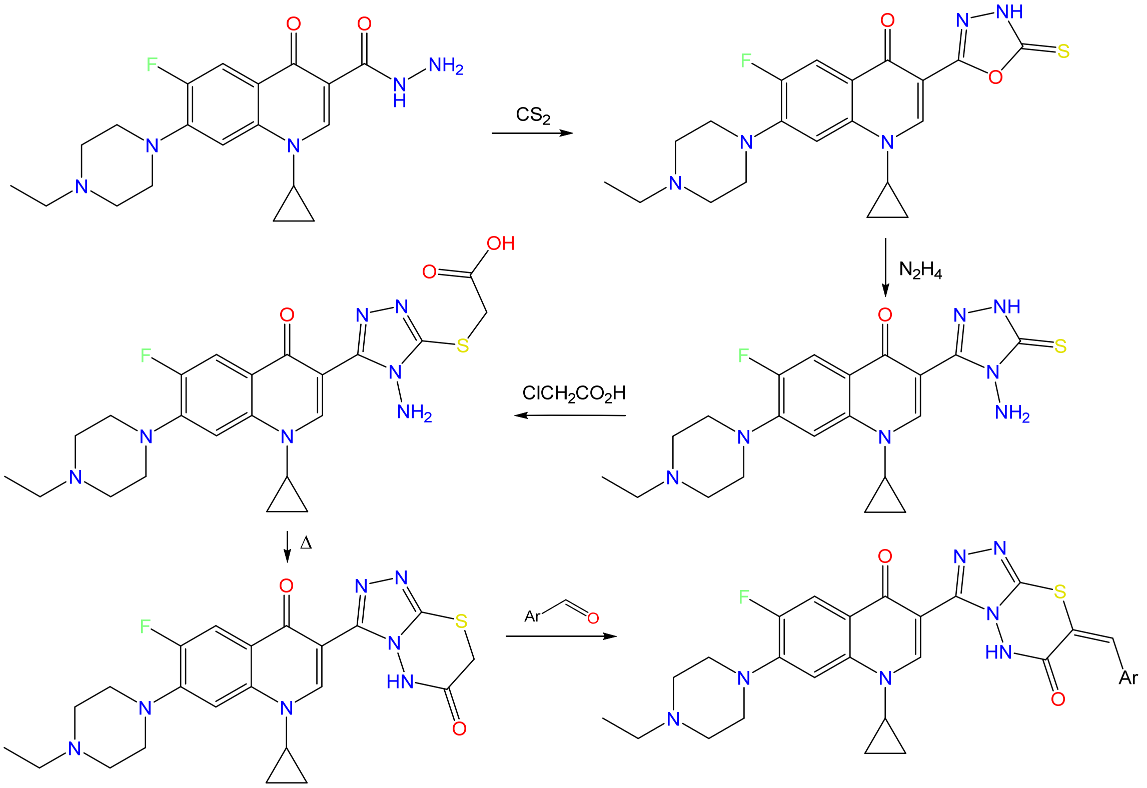

[51] Nosova, E. V.; Sidorova, L. P.; Lipunova, G. N.; Mochul'skaya, N. N.; Chasovskikh, O. M.; Charushin, V. N. Synthesis of new fluorinated derivatives of quinolinecarboxylic acids. *Chemistry of Heterocyclic Compounds*, **2002**, 38(8), 922-928

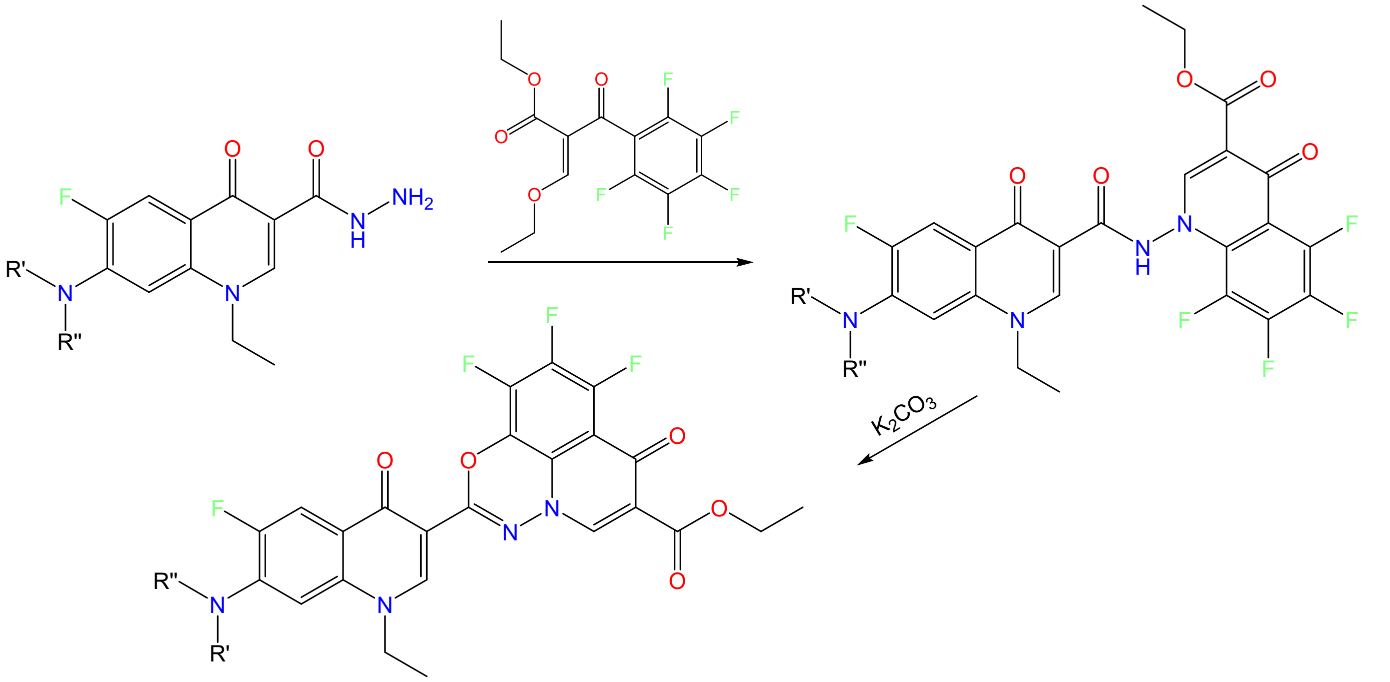

[52] Hu, Guoqiang; Yang, Yong; Yi, Lei; Wang, Xin; Zhang, Zhiqiang; Xie, Songqiang; Huang, Wenlong Design, synthesis and antitumor action of C3/C3 bis-fluoroquinolones linked-cross 2,5-[1,3,4]oxadiazole. *Yaoxue Xuebao*, **2010,** 45(8), 1012-1016

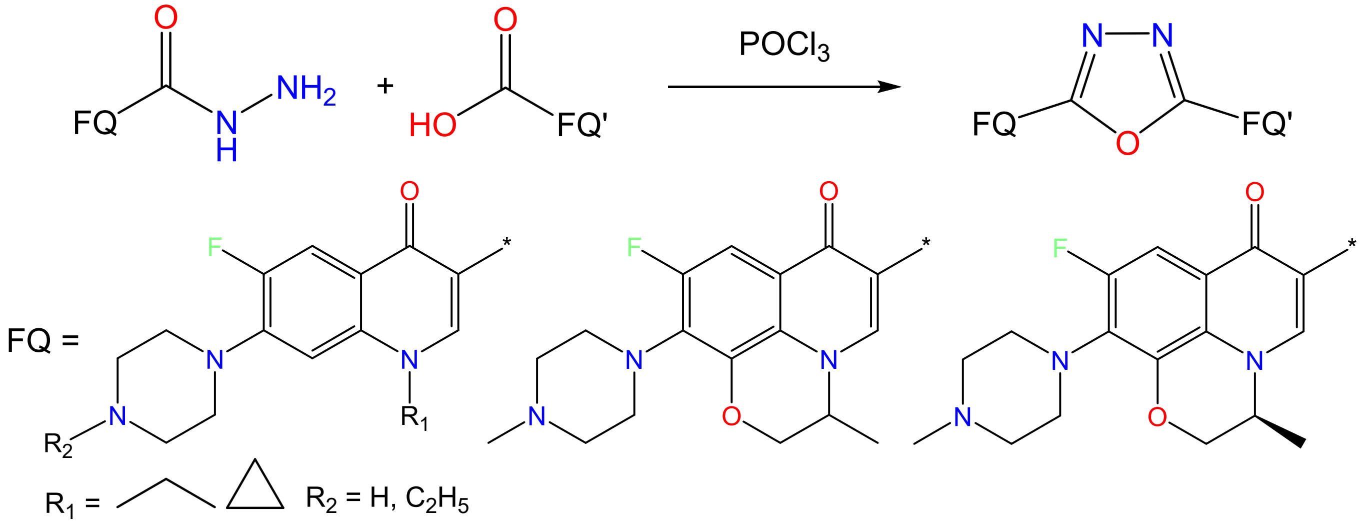

[53] Hu, Guo-Qiang; Yang, Yong; Yi, Lei; Wang, Guo-Qiang; Duan, Nan-Nan; Wen, Xiao-Yi; Cao, Tie-Yao; Xie, Song-Qiang; Huang, Wen-Long Design, synthesis and antitumor activity of C3/C3 bis-fluoroquinolones cross-linked with [1,2,4]triazolo[3,4-b][1,3,4]thiadiazole. *Acta Pharmaceutica Sinica B*, **2011,** 1(3), 172-177

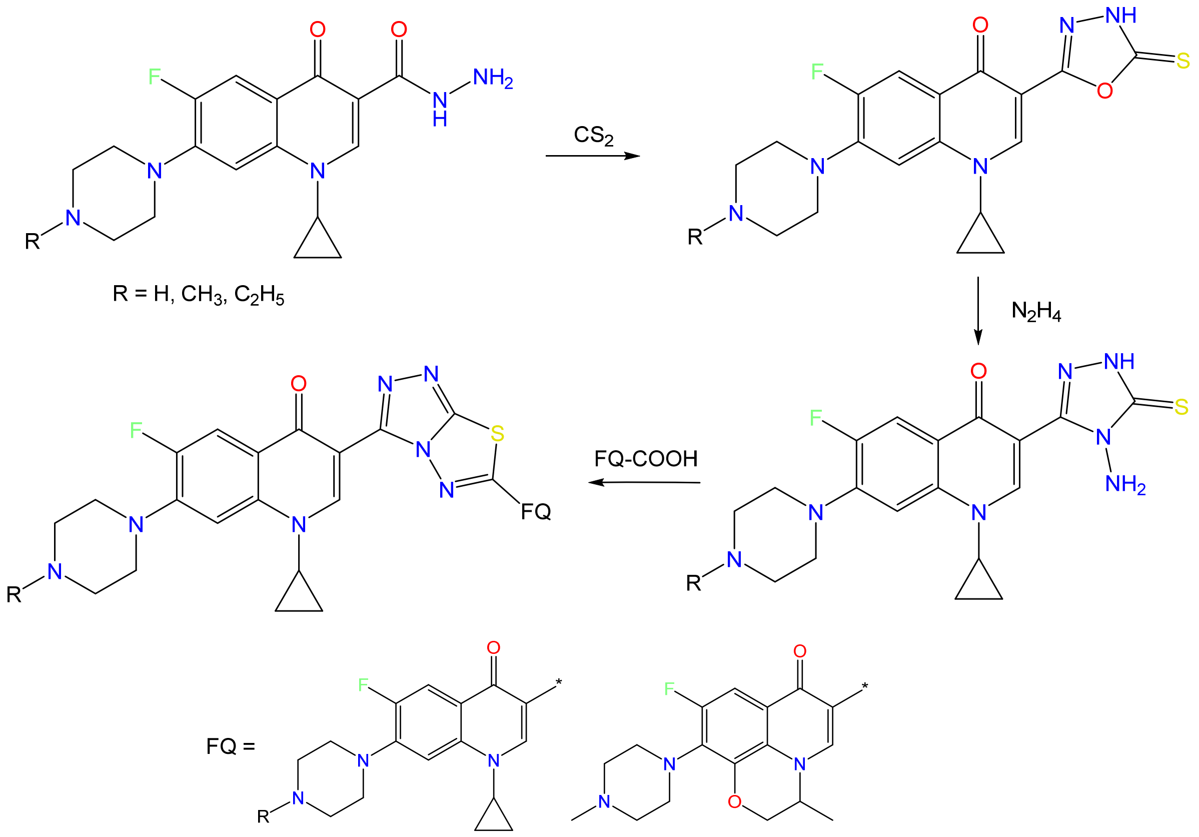

[54] Hu, Guo Qiang; Zhang, Zhong Quan; Xie, Song Qiang; Huang, Wen Long Synthesis and antitumor evaluation of C3/C3 fluoroquinolone dimers (I), tethered with a fused heterocyclic s-triazolo[2,1-b][1,3,4]thiadiazole. *Chinese Chemical Letters*, **2010**, 21(6), 661-663

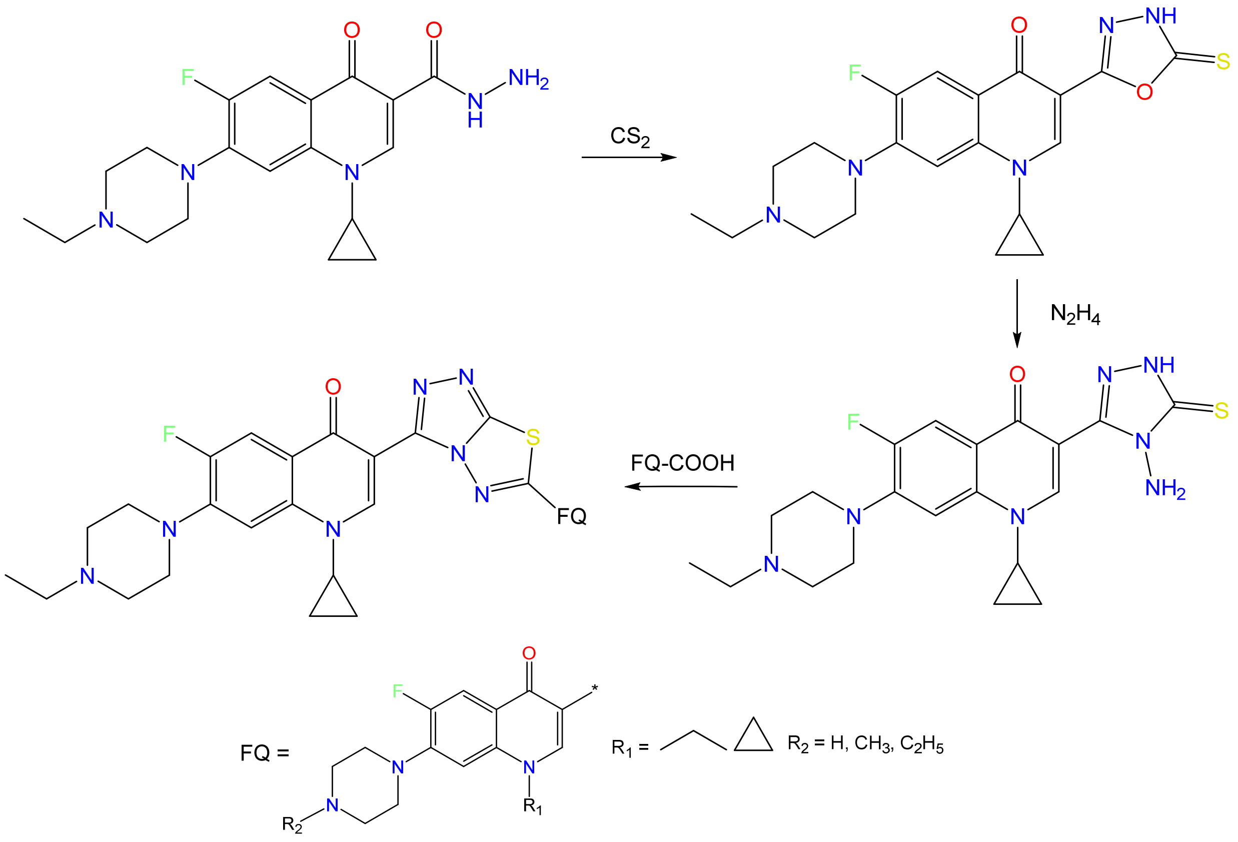

[56] Li, Bei; Cociorva, Oana M.; Nomanbhoy, Tyzoon; Li, Qiang; Nakamura, Kai; Nomura, Masahiro; Okada, Kyoko; Yumoto, Kazuhiro; Liyanage, Marek; Zhang, Melissa C.; et al 6-Position optimization of tricyclic 4-quinolone-based inhibitors of glycogen synthase kinase-3β: Discovery of nitrile derivatives with picomolar potency. *Bioorganic & Medicinal Chemistry Letters,* **2012**, 22(2), 1005-1008

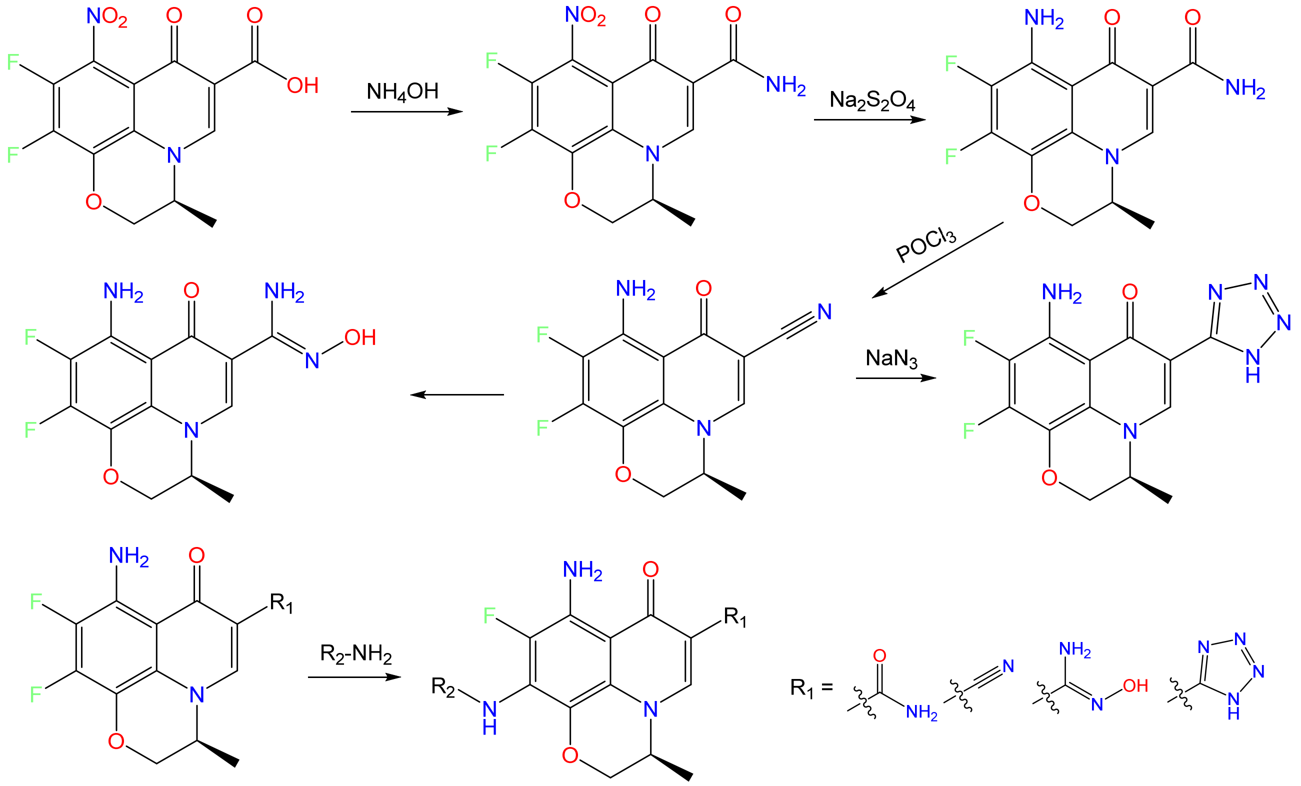

[57] Kumar, Dange V.; Rai, Roopa; Brameld, Ken A.; Riggs, Jennifer; Somoza, John R.; Rajagopalan, Ravi; Janc, James W.; Xia, Yu M.; Ton, Tony L.; Hu, Huiyong; et al 3-Heterocyclyl quinolone inhibitors of the HCV NS5B polymerase *Bioorganic & Medicinal Chemistry Letters,* **2012**, 22(1), 300-304

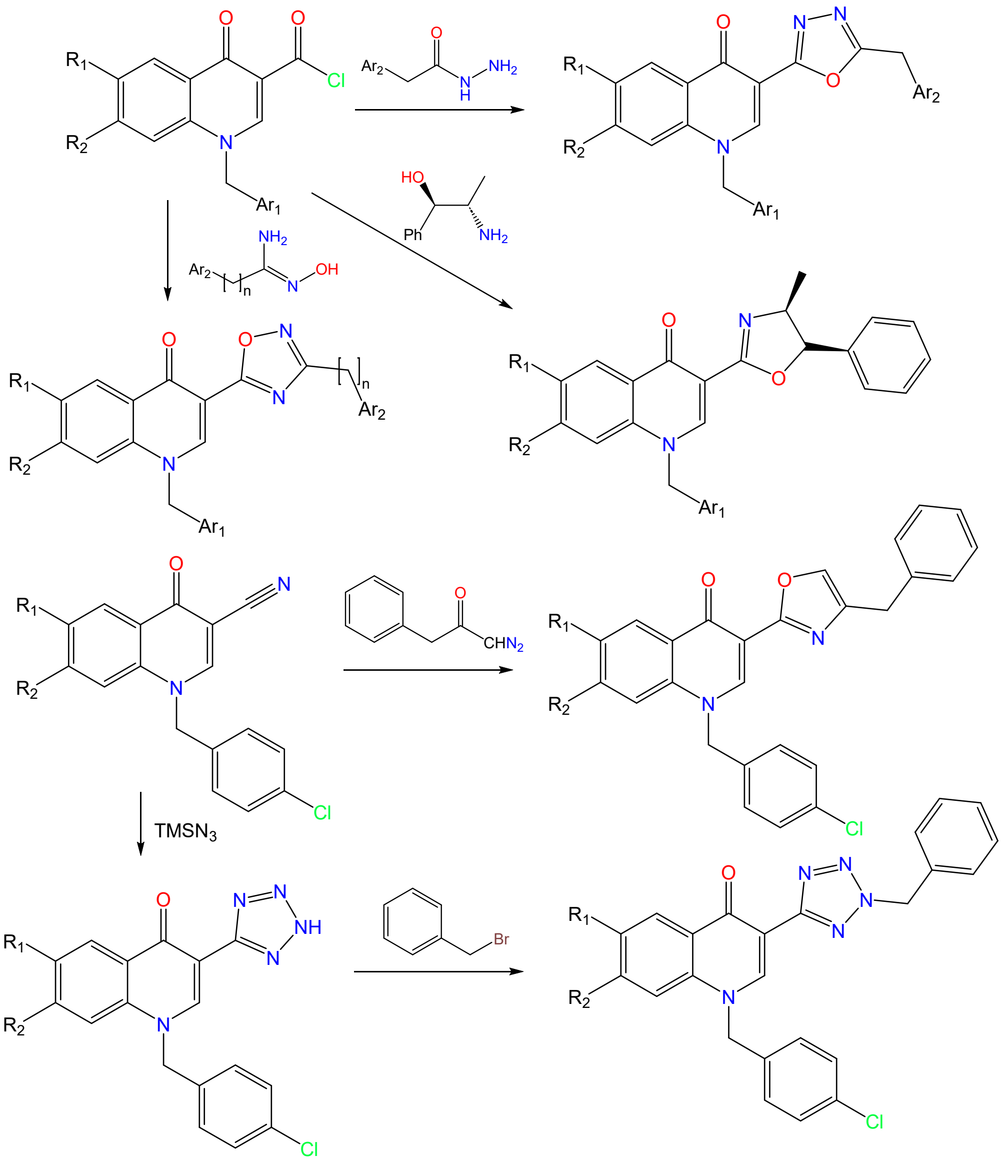

[58] Shaikh, Asif R.; Patel, Navin B.; Rajani, Dhanji Antimycobacterial and antimicrobial studies of newly synthesized 3-(4-(6-methylbenzo[d]thiazol-2-yl)phenyl)quinazolin-4(3H)-ones. *Indian Journal of Research in Pharmacy and Biotechnology,* **2014,** 2(1), 935-942, 8 pp.

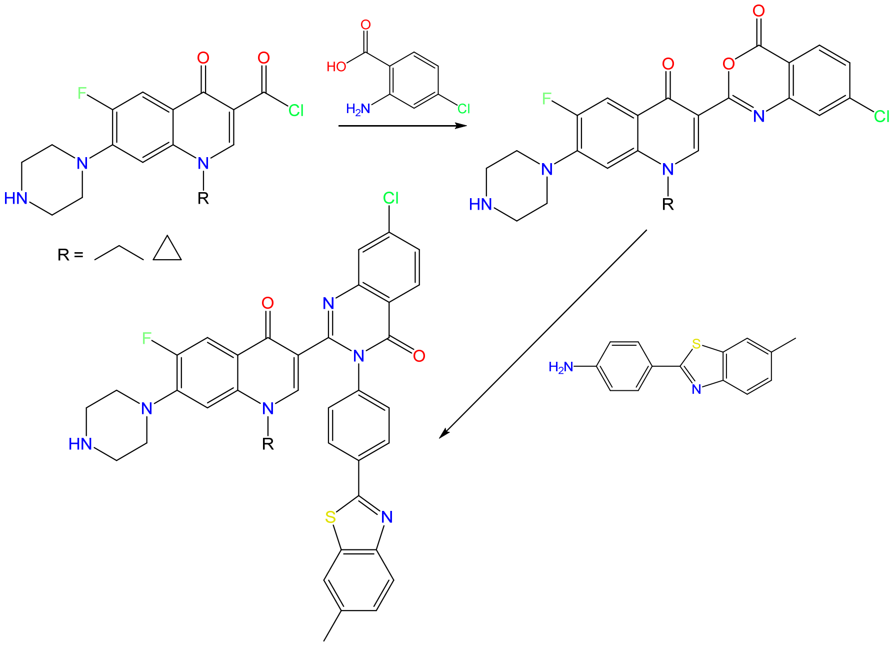

[59] Pyrih, Andriy; Berninger, Michael; Gzella, Andrzej; Lesyk, Roman; Holzgrabe, Ulrike Synthesis and evaluation of antitrypanosomal activity of some thiosemicarbazide derivatives of 1-butyl-6-fluoro-7-morpholino-4-oxo-1,4-dihydroquinoline-3-carboxylic acid. *Synthetic Communications*, **2018**, 48(14), 1883-1891

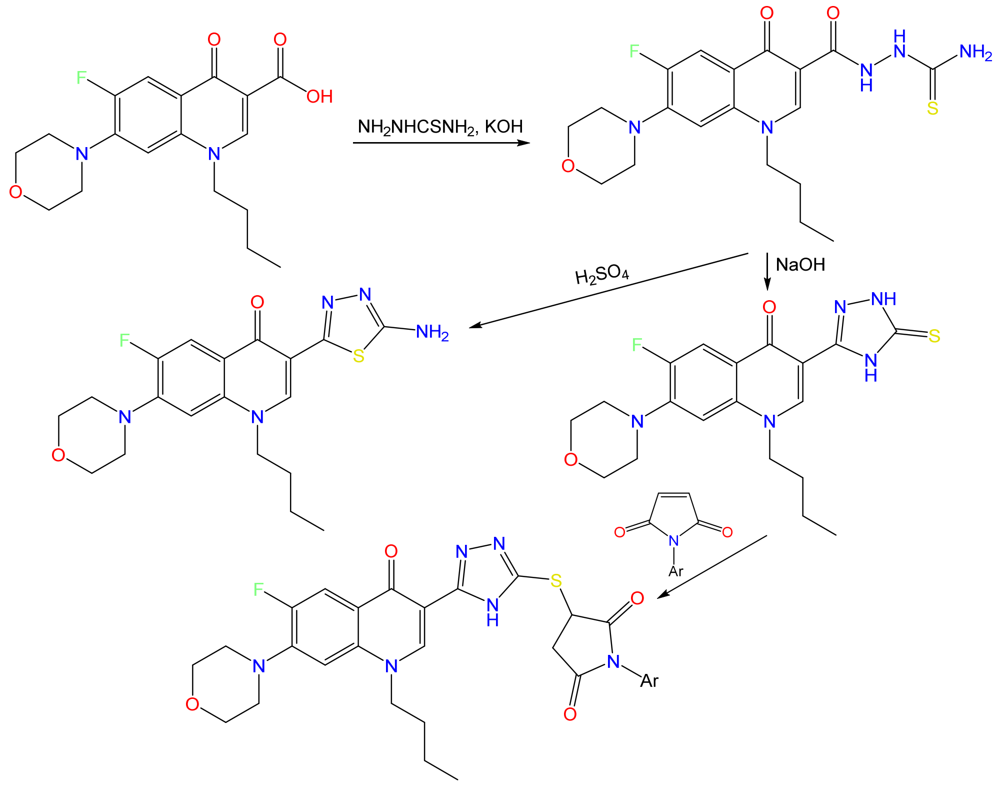

[60] Gao, Liu-zhou; Li, Tao; Xie, Yu-suo; Hu, Guo-qiang; Huang, Wen-long Synthesis and antitumor activity of fluoroquinolon-3-yl s-triazole sulfanylacetylhydrazones and s-triazole hydrazone derivatives (V). *Zhongguo Yaoxue Zazhi (Beijing, China)*, **2015**, 50(6), 545-549

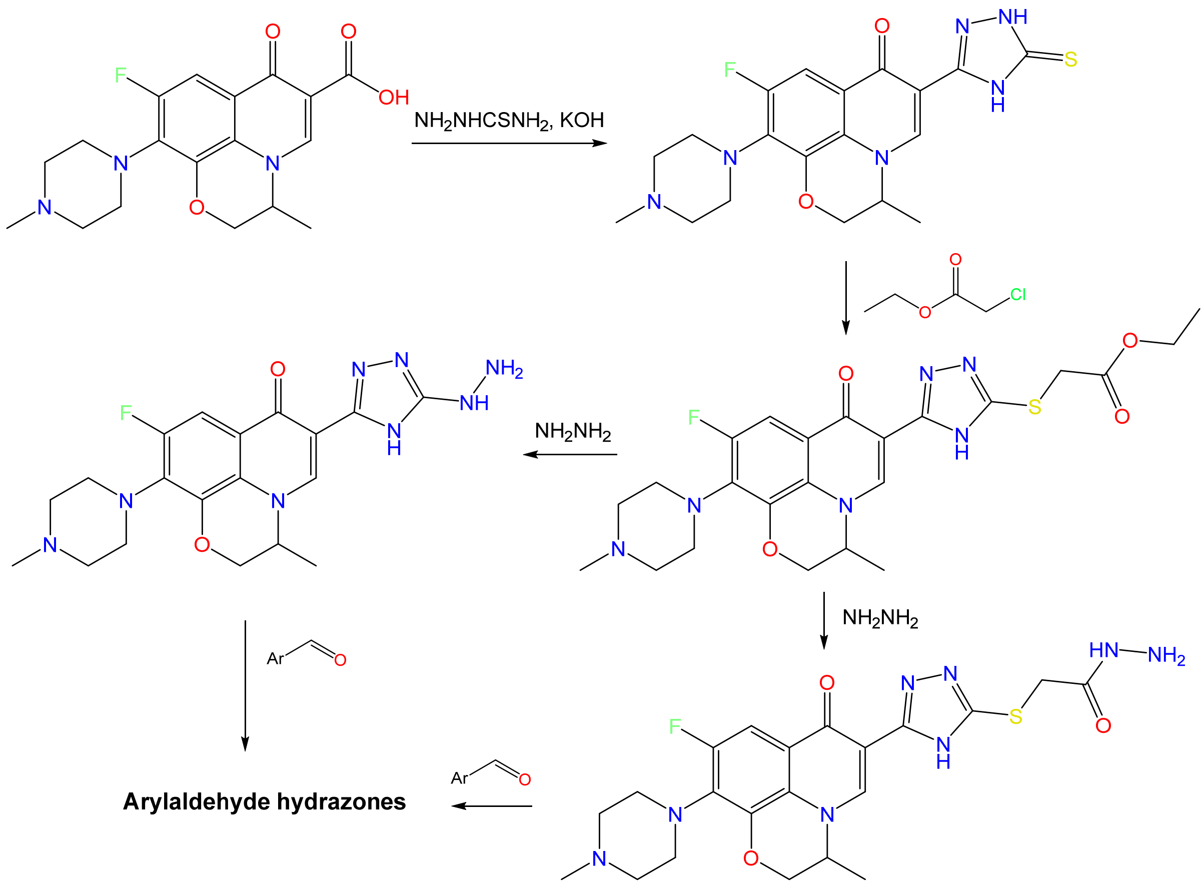

[61] Wang, Liang-Liang; Battini, Narsaiah; Bheemanaboina, Rammohan R. Yadav; Zhang, Shao-Lin ; Zhou, Cheng-He Design and synthesis of aminothiazolyl norfloxacin analogues as potential antimicrobial agents and their biological evaluation. *European Journal of Medicinal Chemistry*, **2019,** 167, 105-123

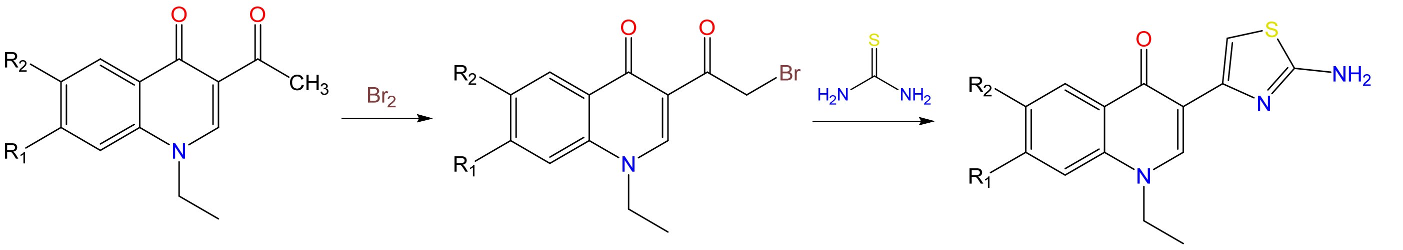

[62] Cui, Sheng-Feng; Addla, Dinesh; Zhou, Cheng-He Novel 2-aminothiazolylquinolones: design, synthesis, bioactive evaluation, SARs, and preliminary antibacterial mechanism. *Journal of Medicinal Chemistry*, **2016**, 59(10), 4488-4510

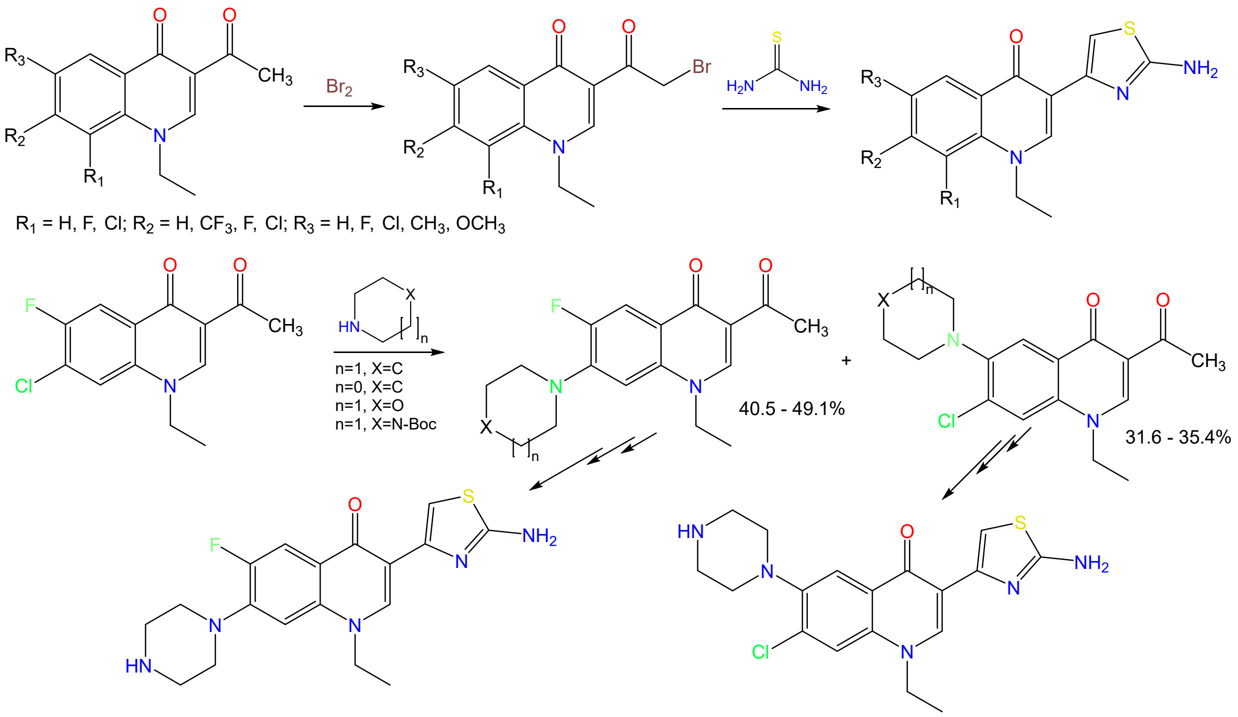

[63] Azad, Chandra S.; Narula, Anudeep K. An operational transformation of 3-carboxy-4-quinolones into 3-nitro-4-quinolones via ipso-nitration using polysaccharide supported copper nanoparticles: synthesis of 3-tetrazolyl bioisosteres of 3-carboxy-4-quinolones as antibacterial agents. *RSC Advances*, **2016**, 6(23), 19052-19059

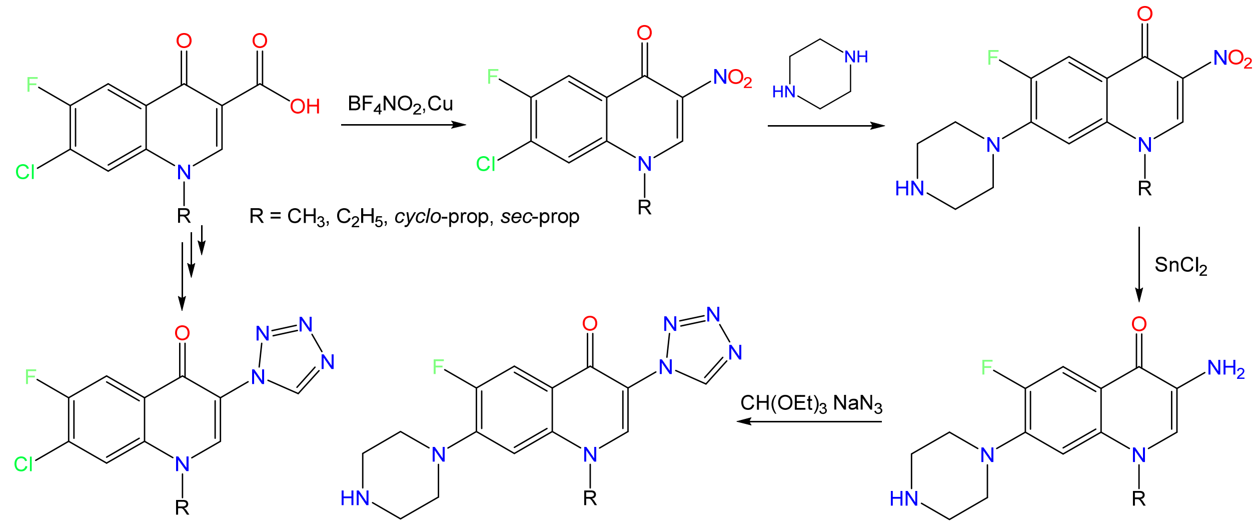

[64] Patel, Navin B.; Shaikh, Asif R.; Soni, Hetal I.; Parmar, Rahul B.; Patel, Jaydeep A. In vitro antimicrobial, antimycobacterial evaluation and synthesis of substituted 1,2,4-triazole motifs. *Chemistry&Biology Interface*, **2018**, 8(3), 184-193

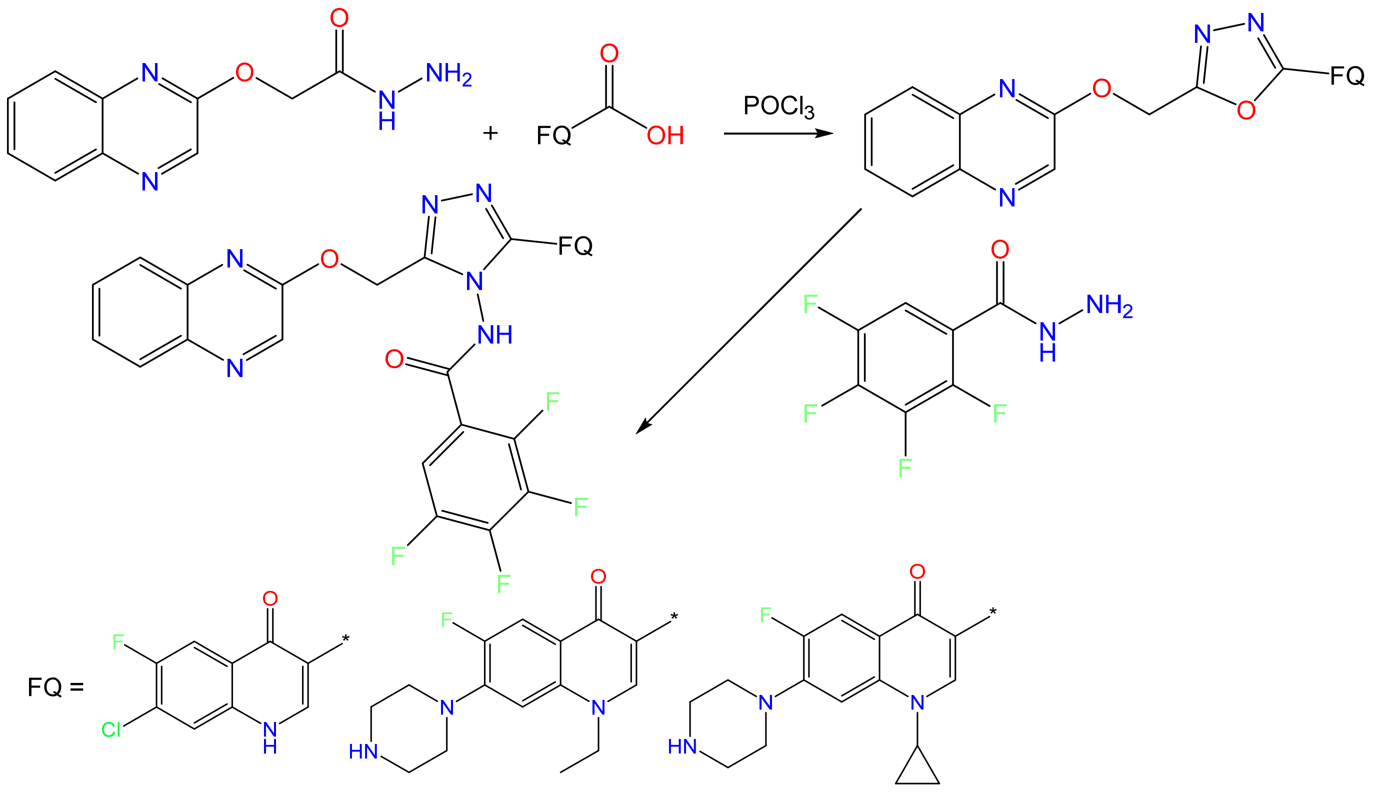

[65] Jubie, S.; Kalirajan, R.; Yadav, Pavankumar Design, synthesis and docking studies of a novel ciprofloxacin analogue as an antimicrobial agent. *Journal of Chemistry*, **2012**, 9(2), 980-987

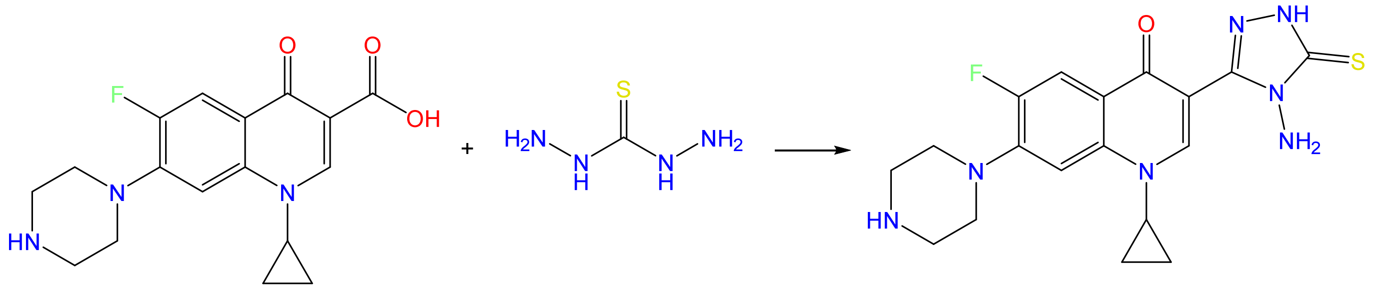

[66] Shaharyar, Mohammad; Ali, Mohamed Ashraf; Abdullah, Mohammad Mustaqeem Synthesis and antiproliferative activity of 1-[(sub)]-6-fluoro-3-[(sub)]-1,3,4-oxadiazol-2-yl-7-piperazino-1,4-dihydro-4-quinolinone derivatives. *Medicinal Chemistry Research*, **2007,** 16(6), 292-299

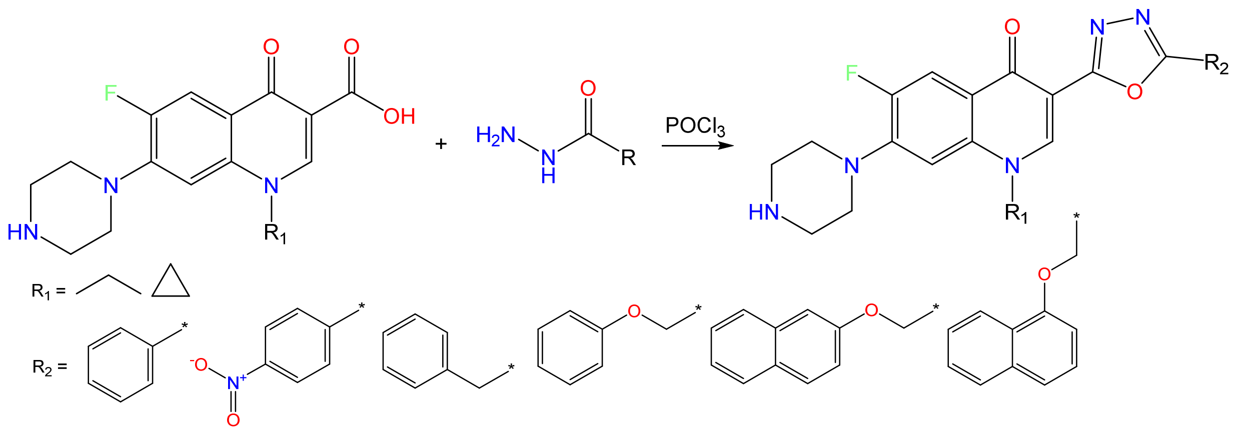

[70] Hu, Guo-qiang; Hou, Li-li; Wang, Guo-qiang; Duan, Nan-nan; Wen, Xiao-yi; Cao, Tie-yao; Yin, Jun; Wang, Wei; Xie, Song-qiang; Huang, Wen-long Design, synthesis and antitumor activity of fluoroquinolone C-3 heterocycles: bis-oxadiazole methylsulfide derivatives derived from ciprofloxacin. *Yaoxue Xuebao*, **2012**, 47(8), 1017-1022

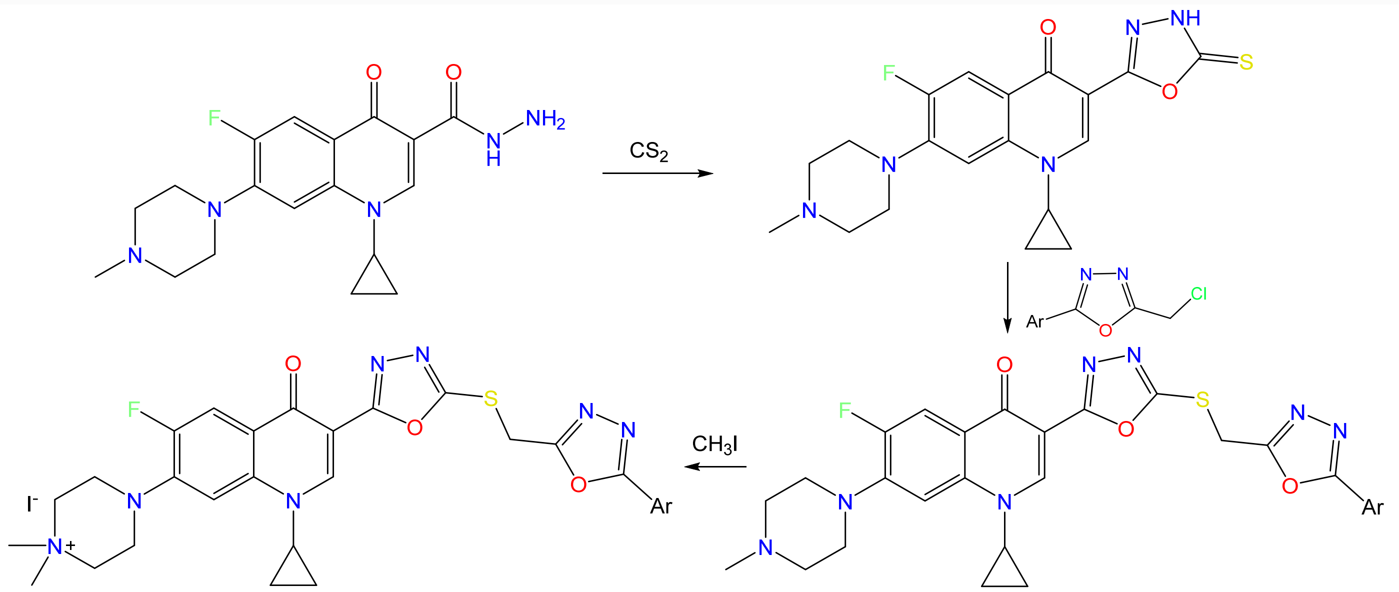

[71] Al-Mathkuri T. S. F, Al-Jubori H. M. S, Saleh A. T. Synthetic and Study the Chelating Activity of Some Polymers Containing Heterocyclic Rings Which Derivative from 1,2,4- Trizol Levofloxacin Acid. *Orient J Chem*, **2018**; 34(4).

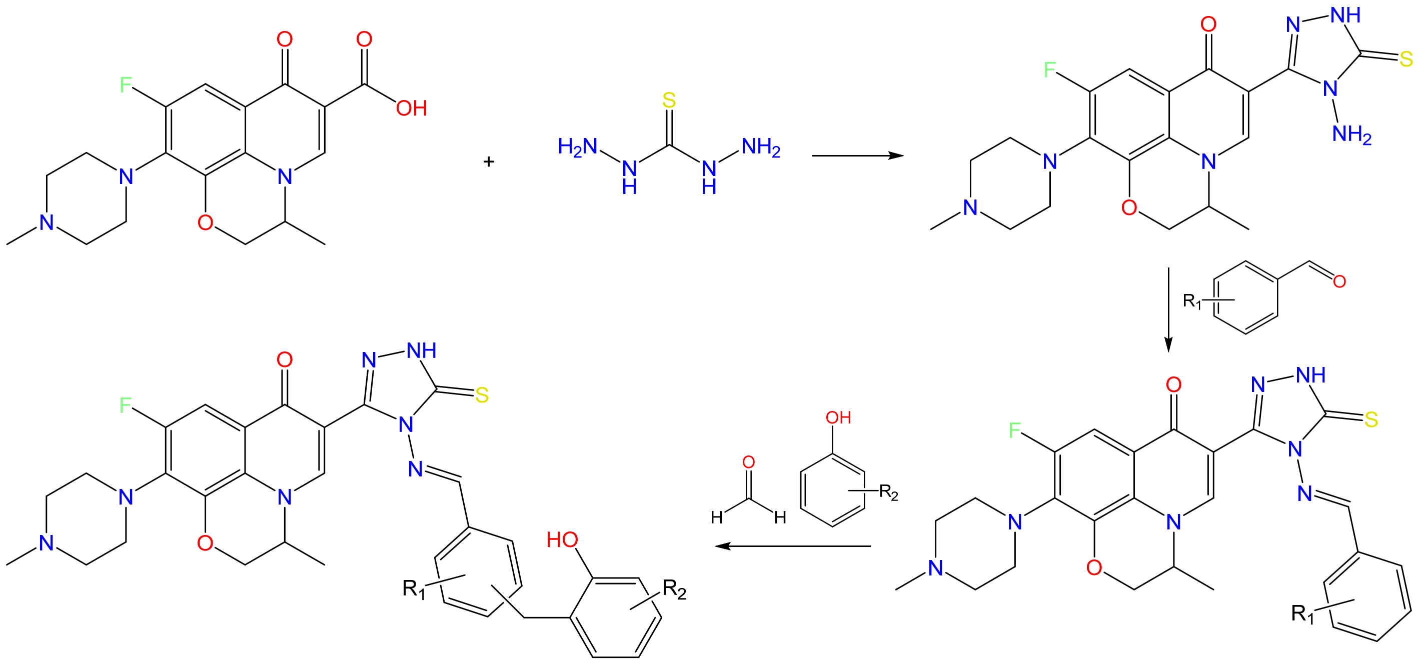

[77] Hu, Guoqiang; Wu, Xiaokui; Wang, Xin; Zhang, Zhiqiang; Xie, Songqiang; Huang, Wenlong; Zhang, Huibin Synthesis and antitumor activity of C3 heterocyclic-substituted fluoroquinolone derivatives (I): ciprofloxacin aminothiodiazole Schiff-bases. *Yaoxue Xuebao*, **2008**, 43(11), 1112-1115

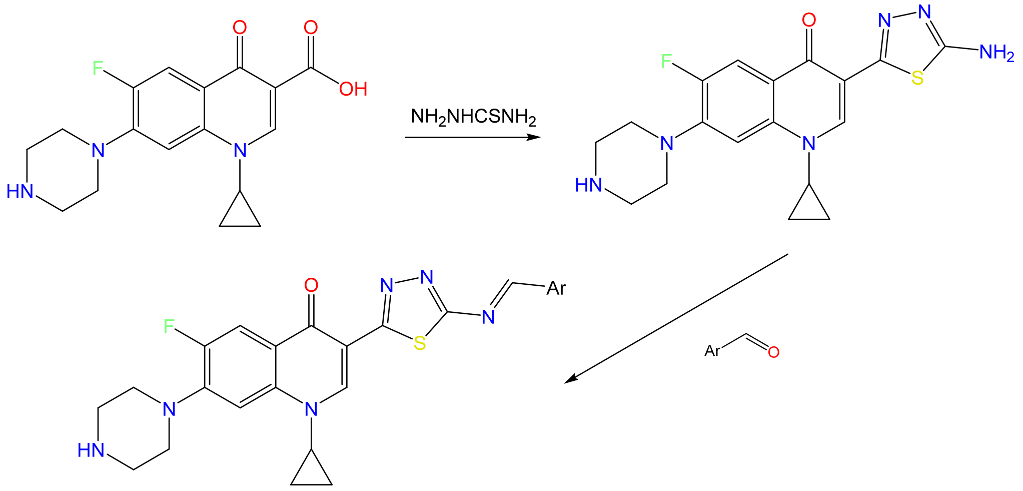

[79] Farooqi, Shahid Iqbal; Arshad, Nasima; Channar, Pervaiz Ali; Perveen, Fouzia; Saeed, Aamer; Larik, Fayaz Ali; Javeed, Aneela Synthesis, theoretical, spectroscopic and electrochemical DNA binding investigations of 1, 3, 4-thiadiazole derivatives of ibuprofen and ciprofloxacin: Cancer cell line studies. *Journal of Photochemistry and Photobiology, B: Biology*, **2018**, 189, 104-118

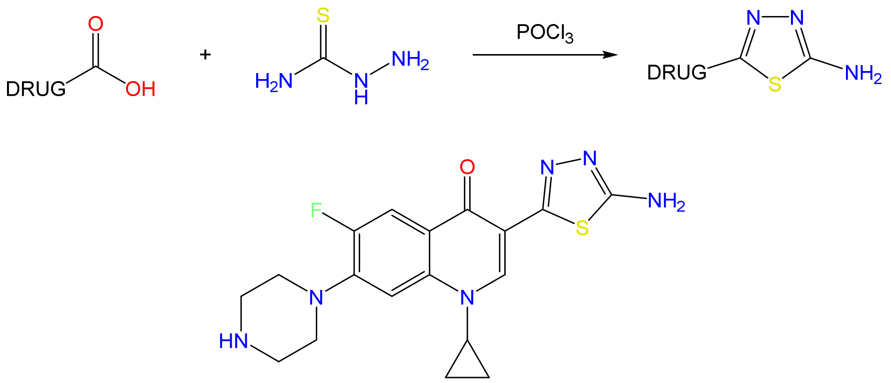

[81] Ujan, Rabail; Saeed, Aamer; Channar, Pervaiz Ali; Larik, Fayaz Ali; Abbas, Qamar; Alajmi, Mohamed F.; El-Seedi, Hesham R.; Rind, Mahboob Ali; Hassan, Mubashir; Raza, Hussain ; et al Drug-1,3,4-thiadiazole conjugates as novel mixed-type inhibitors of acetylcholinesterase: synthesis, molecular docking, pharmacokinetics, and ADMET evaluation. *Molecules,* **2019**, 24(5), 860/1-860/14

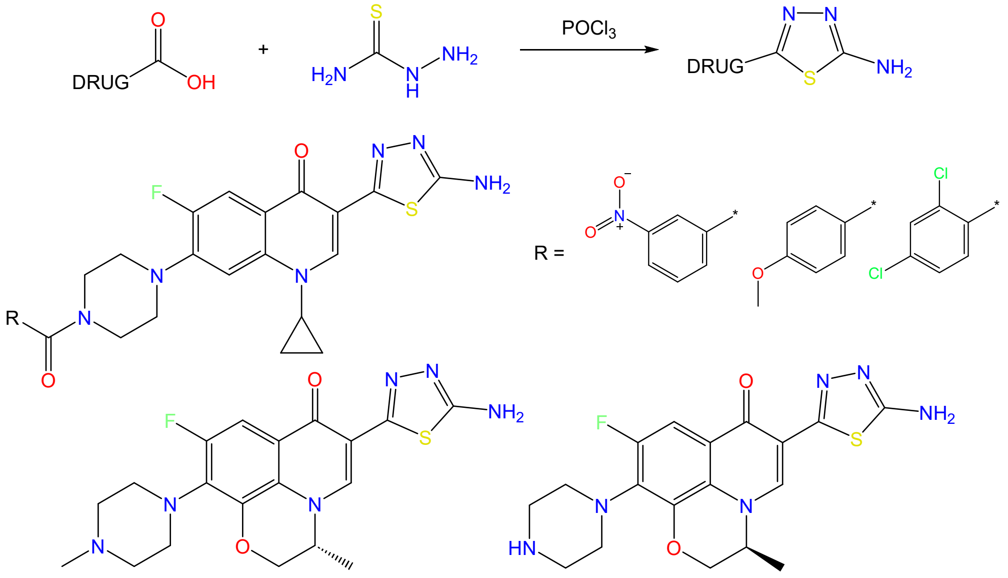

[82] Chandramouli; Shivanand, M. R.; Nayanbhai, Thakar Bhaumik; Bheemachari; Udupi, R. H. Synthesis and biological screening of certain new triazole Schiff bases and their derivatives bearing substituted benzothiazole moiety. *Journal of Chemical and Pharmaceutical Research*, **2012,** 4(2), 1151-1159

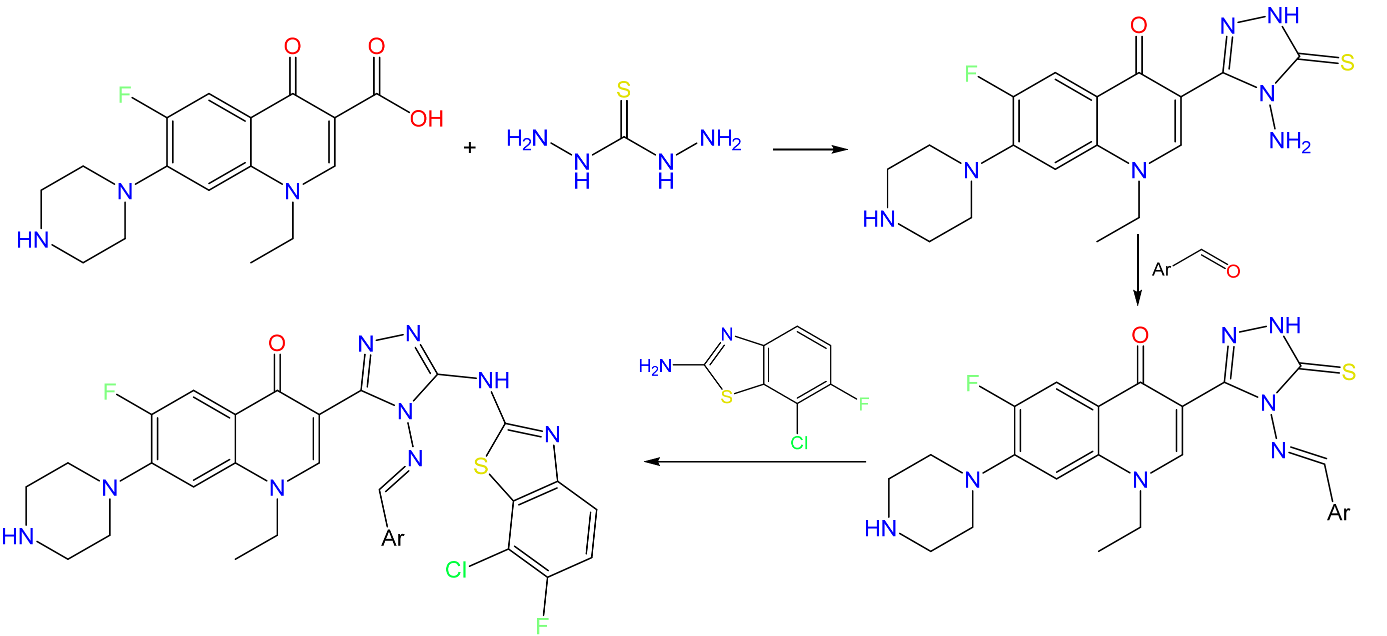

[83] Gao, Liu-zhou; Li, Tao; Xie, Yu-suo; Hu, Guo-qiang; Huang, Wen-long Synthesis and antitumor activity of fluoroquinolon-3-yl s-triazole sulfanylacetylhydrazones and s-triazole hydrazone derivatives (V). *Zhongguo Yaoxue Zazhi (Beijing, China),* **2015**, 50(6), 545-549

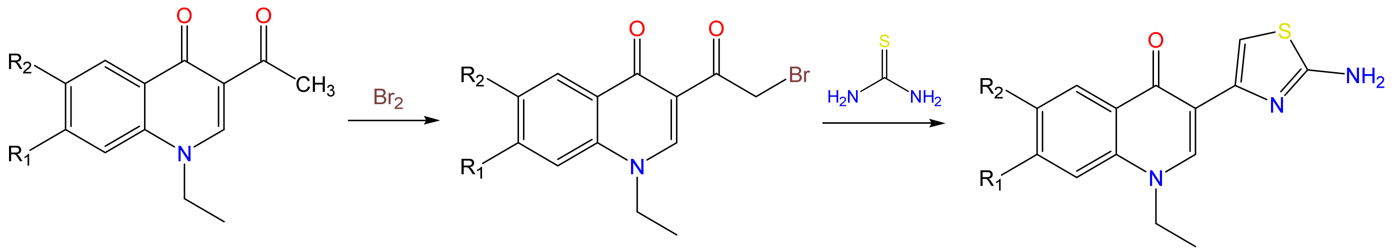

[89] Fokin, A. S.; Burgart, Y. V.; Saloutin, V. I.; Chupakhin, O. N. Synthesis of 2-(1-alkyl(aryl)-4-oxo-5,6,7,8-tetrafluoro-1,4-dihydroquinolin-3-yl)glyoxylic acid derivatives. *Journal of Fluorine Chemistry*, **2001**, 108(2), 187-194

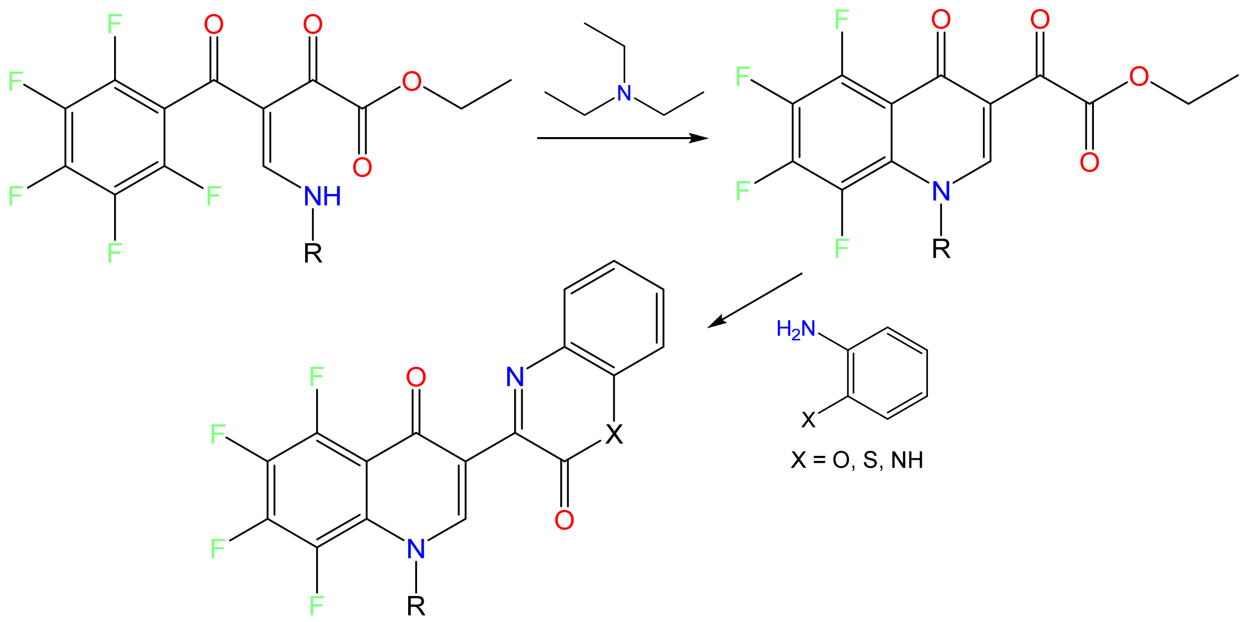

[91] Hu, Guo Qiang; Hou, Li Li; Yang, Yong; Yi, Lei; Xie, Song Qiang; Wang, Guo Qiang; Duan, Nan Nan; Chao, Tie Yao; Wen, Xiao Yi; Huang, Wen Long Synthesis and antitumor evaluation of fluoroquinolone C3 fused heterocycles (II): From triazolothiadiazines to pyrazolotriazoles. *Chinese Chemical Letters*, **2011**, 22(7), 804-806

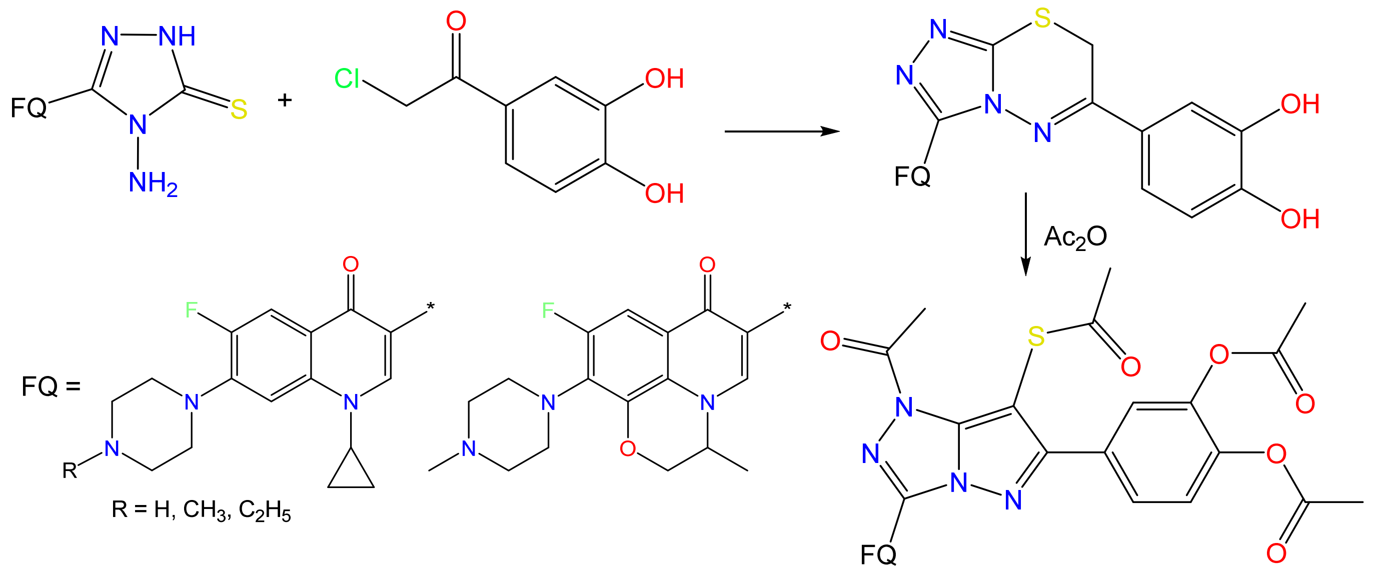

[94] Muluk, Rekha; Kothawade, Prajakta; Kulkarni, Gauri; Ingale, Pramode Synthesis and evaluation of some novel benzimidazole-quinolinone derivatives for their antifungal and antidiabetic activity. *World Journal of Pharmacy and Pharmaceutical Sciences,* **2018**, 7(1), 1263-1278

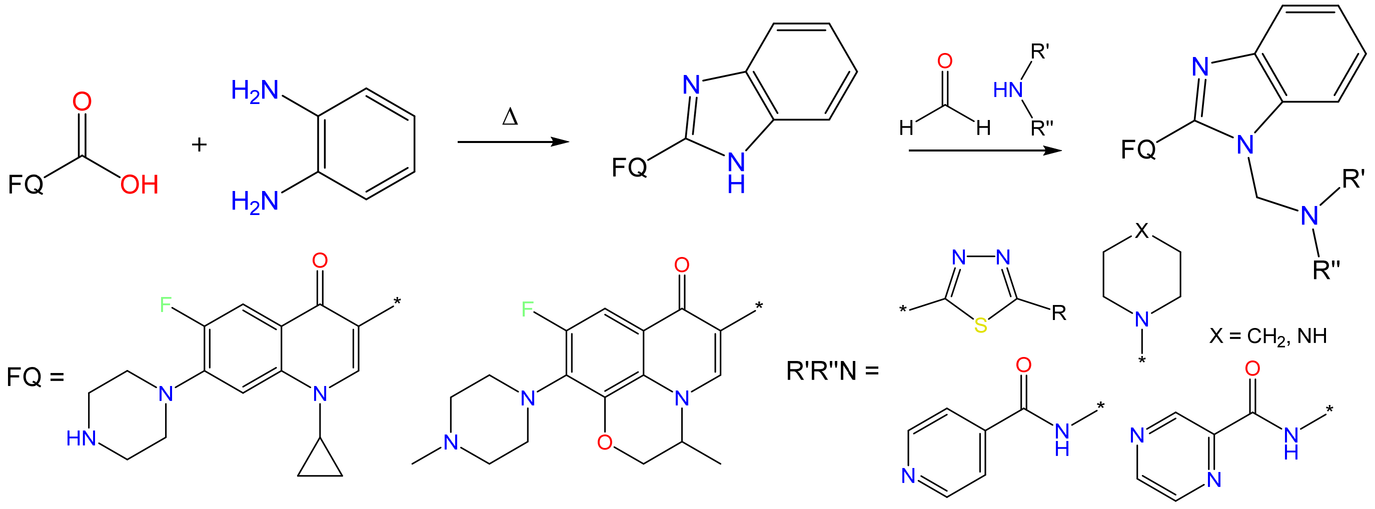

[95] You, Qi-Dong; Li, Zhi-Yu; Huang, Chiung-Hua; Yang, Qian; Wang, Xiao-Jian; Guo, Qing-Long; Chen, Xiao-Guang; He, Xun-Gui; Li, Tsai-Kun; Chern, Ji-Wang Discovery of a Novel Series of Quinolone and Naphthyridine Derivatives as Potential Topoisomerase I Inhibitors by Scaffold Modification. *Journal of Medicinal Chemistry*, **2009,** 52(18), 5649-5661

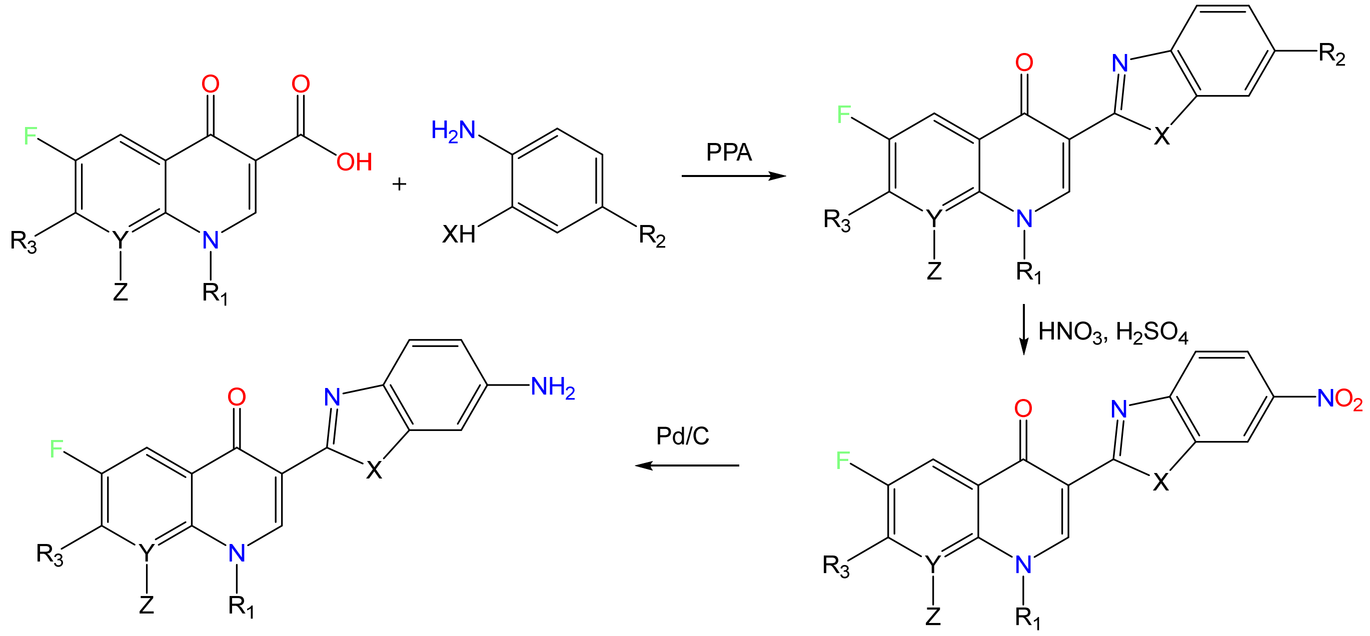

Supplement: Supplementary file 1 [file antibiotics-12-00625-s001.zip › antibiotics-2280726-supplementary.pdf]
